# Incentive valence differentially engages open- and closed-loop basal ganglia circuits during movement initiation

**DOI:** 10.1073/pnas.2537314123

**Published:** 2026-05-06

**Authors:** Neil M. Dundon, Elizabeth J. Rizor, Joanne E. Stasiak, Jingyi Wang, Taylor Li, Kiana Sabugo, Christina Villanueva, Parker Barandon, Viktoriya Babenko, Renee Beverly-Aylwin, Alexandra Stump, Tyler Santander, Andreea C. Bostan, Regina C. Lapate, Scott T. Grafton

**Affiliations:** ^a^Department of Psychological and Brain Sciences, University of California, Santa Barbara, CA 93106; ^b^Aligning Science Across Parkinson’s Collaborative Research Network, Chevy Chase, MD 20815; ^c^ Department of Social and Psychological Sciences, University of Huddersfield, Huddersfield HD1 3DH, United Kingdom; ^d^Department of Neuroscience, University of California, Berkeley, CA 94720; ^e^ Department of Psychology and Neuroscience, Duke University, Durham, NC 27708; ^f^ BIOPAC Systems, Inc., Goleta, CA 93117; ^g^ Department of Medicine, College of Medicine Tucson, University of Arizona, Tucson, AZ 85724; ^h^Department of Neurobiology, University of Pittsburgh School of Medicine, Pittsburgh, PA 15213

**Keywords:** motivation, motor control, striatum, functional connectivity, Parkinson’s disease

## Abstract

Incentives modulate voluntary movement, yet the circuitry channeling these signals into motor output remains unclear. Classical models emphasize a closed-loop circuit (CLC) linking dorsal putamen (PUTd) with the motor cortex, but this pathway is anatomically segregated from affective processing regions. Anatomical and clinical evidence point to an alternative: an open-loop circuit (OLC) from ventral putamen (PUTv) that may route affective signals to the motor cortex. Here, we conducted two experiments to test whether a functional OLC exists in humans and whether it is differentially engaged by incentive conditions. First, in 7 T resting-state fMRI (multi-echo), PUTv showed robust functional connectivity with both affective and motor regions, including the cingulate motor area, even after accounting for PUTd variance. This connectivity pattern supports the plausibility of an independent pathway linking affective basal ganglia regions to the motor cortex. Second, in 3 T task fMRI (incentivized reaching), jackpot (high-reward) and robber (high-loss avoidance) incentive conditions produced distinct behavioral and neural signatures. Jackpot produced a speed–accuracy trade-off, with faster movement initiation but more false starts. Neurally, this coincided with reduced engagement (BOLD responses relevant for initiation speed) in CLC nodes but not in OLC. Robber, in contrast, eliminated engagement in both OLC and CLC nodes, instead recruiting stopping-related regions (e.g., subthalamic nucleus), consistent with an avoidance phenomenology. Together, these findings support a versatile architecture for movement initiation that flexibly engages distinct cortico-subcortical circuits depending on incentive phenomenology, and offer a candidate mechanism through which affective salience and valence modulate voluntary movement.

## Significance

Affective signals profoundly influence movement, yet the mechanisms linking motivationally relevant contexts with motor behavior remain unclear. Combining ultra-high-field (7 T) connectomics with task-based (3 T) neuroimaging, we provide systems-level evidence in humans for such a mechanism: a ventral putamen-centered open-loop circuit connecting affective and motor areas, operating alongside the canonical dorsal putamen-centered closed-loop sensorimotor circuit. Critically, the phenomenological quality of incentive (how it is construed as reward vs. threat) rather than magnitude alone, likely determines which circuit dominates during movement initiation. These findings help to explain paradoxical kinesia in Parkinson’s disease, where affective contexts can bypass degraded sensorimotor circuits, and establish foundations for context-based therapeutic interventions.

Control and execution of voluntary movement is sensitive to a host of contextual factors including affective and reward-driven incentive (1–5). Both cortical and subcortical regions, including the basal ganglia and amygdala, play a role in processing affective and reward information, suggesting an indirect influence over motor cortical regions (6). However, the mechanisms by which affective signals are integrated into movement remain unclear. Traditional neuroanatomical understanding of basal ganglia interactions with the motor cortex (7, 8) in mammals centers on a canonical closed-loop circuit (CLC) entering the basal ganglia via the dorsal (“sensorimotor”) putamen (PUTd), projecting through globus pallidus internus (GPi), and returning to primary motor cortex (M1) via ventrolateral thalamus (VL). This sensorimotor CLC is anatomically and functionally distinct from other CLCs that link the basal ganglia with cognitive and affective regions of the cerebral cortex (7). The CLC plays a crucial role in voluntary movement, as underscored by the impairments following loss of dopaminergic innervation of PUTd (9) in Parkinson’s disease (PD), which include slowness and difficulty in initiating movement (10).

**Fig. 1.**
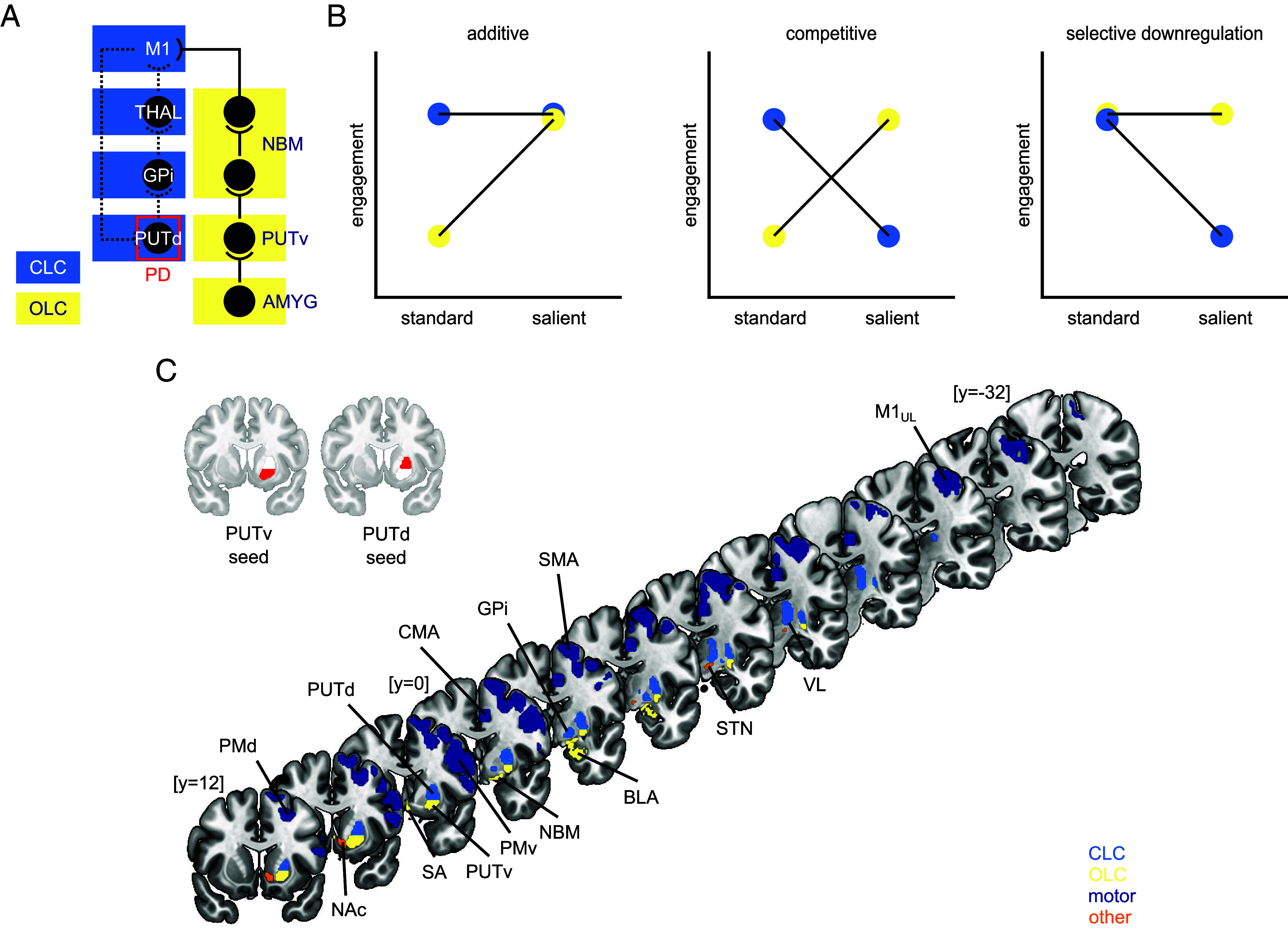
(*A*) Canonical CLC and putative OLC, with site of disruption in PD. (*B*) Three hypothesized patterns of circuit engagement by incentive salience: additive, competitive, and selective downregulation. (*C*) Anatomical regions of interest used across experiments, comprising OLC, CLC, and motor-cortical nodes. Two additional subcortical regions (NAc, STN), grouped as “other,” were included in Experiment 2 only. Anatomical abbreviations are defined in *SI Appendix*, Tables S1 and S2.

Yet, clinical observations suggest that while central, the CLC may not be the sole pathway supporting movement initiation. For instance, although the CLC is impaired in PD, affectively salient events such as car accidents (11), natural disasters (12, 13), and incentives (14) can transiently normalize movement initiation (15), suggesting alternative pathways through which affective signals might influence movement initiation in motivationally relevant contexts. One candidate circuit has been proposed by results from neuroanatomical tracing studies in nonhuman primates (NHPs). Retrograde transneuronal transport of rabies virus from injections in the primary motor cortex (M1) in NHPs shows that both PUTd and the ventral (“limbic”) putamen (PUTv) send multisynaptic projections to M1. Notably, while PUTd is a target of M1 input, PUTv does not receive inputs from M1, but from the amygdala and cortical areas involved in affective and incentive-based functions (16, 17). Efferents from PUTv are thought to reach the motor cortex not via the canonical GPi route, but via basal forebrain [substantia innominata/nucleus basalis of Meynert (NBM)], whose cholinergic projections widely innervate the cerebral cortex (18–20). Together, these structures define a putative open-loop circuit (OLC; Fig. 1*A*) through which affective signals may reach the motor cortex via the amygdala, PUTv, and basal forebrain. PUTv also shows spared dopaminergic innervation in primate models of PD (21). In humans with PD, positron emission tomography imaging similarly shows that the PUTv is relatively spared, with the ventrorostral region remaining least affected even in advanced disease (22).

Combined with the clinical observations in PD, these anatomical and imaging findings suggest that PUTv, and its embedding in the putative OLC, might afford affective or incentive-based processes an influence over voluntary movement under motivationally relevant conditions. However, two critical questions remain unanswered: 1) Does the healthy human brain exhibit a functional organization consistent with a PUTv-centered OLC that couples affective and motor systems, independently of PUTd? And 2) can activity in nodes of the putative OLC (particularly PUTv) support movement initiation under varying incentive conditions, and are the CLC and OLC differentially engaged across these conditions?

Prior systems-neuroscience experiments in humans offer key hypotheses about how incentive conditions might modulate control via differential engagement of OLC and CLC nodes. We here test three differential engagement patterns (Fig. 1*B*). Engagement may be “additive”, where incentives recruit OLC alongside CLC to jointly facilitate movement initiation. Such additive recruitment is consistent with cost–benefit frameworks of movement vigor (23), where value and vigor nodes converge on motor effectors to scale vigor according to expected utility. Engagement may further be “competitive”, with incentives recruiting OLC while suppressing CLC, leading to a shift in control. Such a “winner-take-all” pattern is seen during ventral (value) to dorsal (habit) striatal control shift in human addiction (24) and is similarly observed in rodents, where threat drives defensive circuits to dominate and suppress feeding, in contrast to dominant feeding circuits in the absence of threat (25). Alternatively, engagement may reflect “selective downregulation”, where both circuits are typically engaged, but high incentive salience disengages some circuitry in order to optimize for parsimony under immediate demands. Conventionally, this is observed when incentives prune cognitive control networks, increasing speed at the cost of task accuracy (26–28). Importantly, selective downregulation of hyperactive nodes can also aid neurorehabilitation (29, 30), consistent with models of bradykinesia in PD that implicate excessive activity in canonical stopping circuits (31).

Thus, to identify the putative OLC in humans and examine its possible role in movement initiation, we conducted two complementary neuroimaging studies. First, we examined the plausibility of a PUTv-centered OLC using ultra-high-field (7 T) multi-echo resting-state fMRI, leveraging this paradigm’s spatial-resolution and signal-to-noise advantages to assess whether PUTv is reliably functionally connected with both affective and motor regions, distinct from PUTd. Second, we analyzed task-based fMRI from a separate 3 T sample performing an incentivized precision reaching task. The task adopted the valence-and-salience structure of the monetary incentive delay paradigm [MID; (32)] under three incentive conditions (high-reward, high-loss, and standard-reward), but required speeded precision reaches rather than button presses. Using state-of-the-art BOLD signal modeling (33), we examined how activation duration in predefined CLC and OLC nodes (Fig. 1*C*) scaled with movement initiation speed, reasoning that circuits facilitating initiation should show the strongest responses during the fastest movements. We then tested which engagement pattern (Fig. 1*B*) best explained how salience (high-reward vs. standard-reward) and valence (high-reward vs. high-loss) shaped BOLD activation across the two circuits.

## Results

### Experiment 1.

#### Connectomic evidence of PUTv connections with affective and motor regions.

To test the plausibility of a putative PUTv-centered OLC linking affective regions to the motor cortex, 28 healthy human participants (13 female, mean age 29.68 ± 4.78 y) completed a high-resolution resting-state fMRI scan at 7 T. To isolate intrinsic functional connectivity, we combined multi-echo fMRI with physiological monitoring and ICA denoising to selectively retain BOLD signal (*Materials and Methods*).

Using PUTv and PUTd seeds, we compared their respective connectivity profiles with anatomically defined targets. Our a priori analysis targets included: 1) affective OLC nodes motivated by NHP tracing anatomy: amygdala [basolateral amygdala (BLA), central nucleus of the amygdala (CeA)] as afferents to PUTv, and basal forebrain [NBM, septal area (SA)] as the putative relay to motor cortex (18–20); 2) motor cortical areas [M1, supplementary motor area (SMA), cingulate motor area (CMA), dorsal premotor cortex (PMd), ventral premotor cortex (PMv)]; and 3) CLC output nodes (GPi and VL), included to control for canonical sensorimotor circuit variance (Fig. 1).

**Fig. 2.**
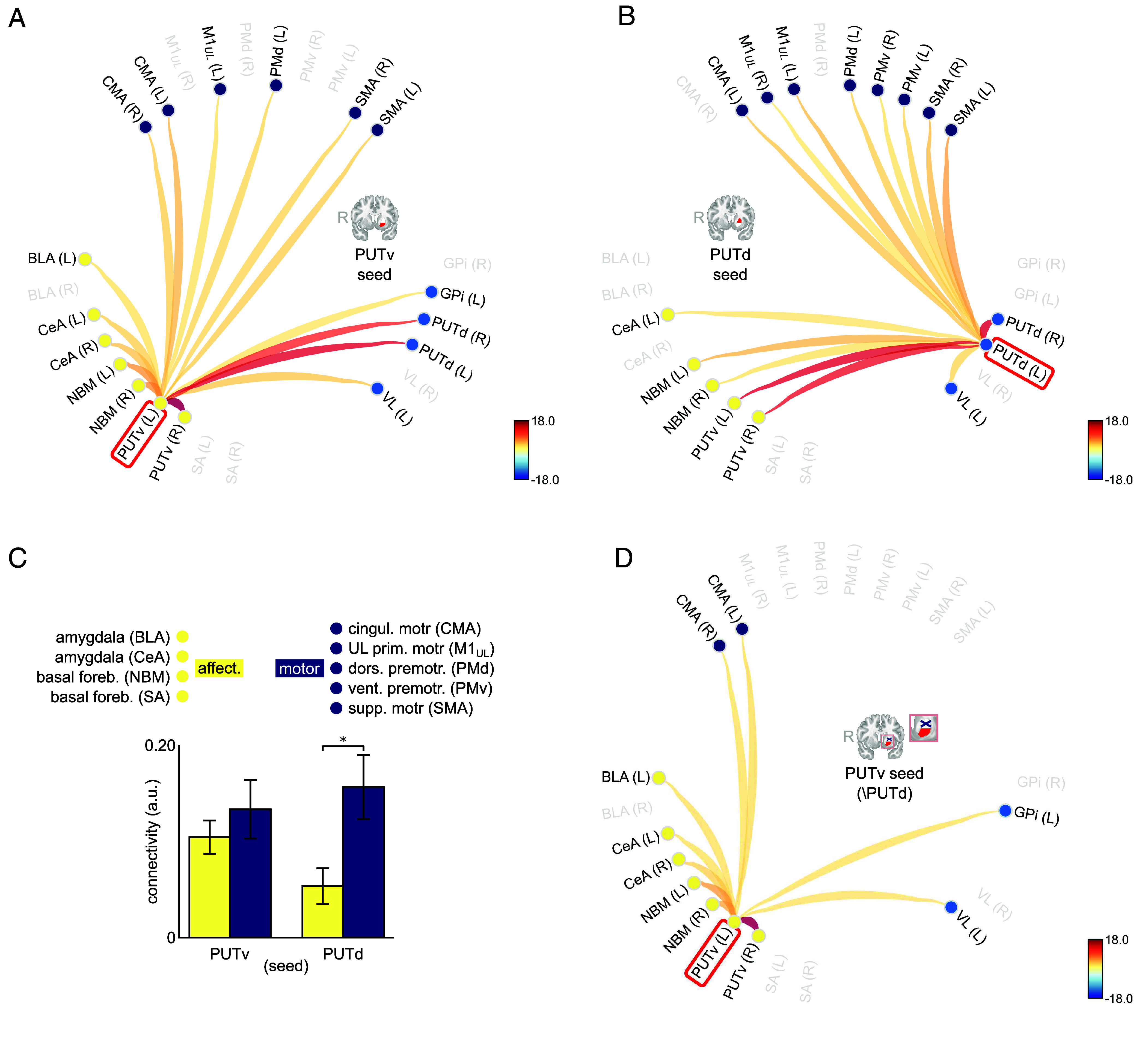
(*A*) Left PUTv connectivity with remaining OLC (yellow), CLC (light blue), and motor-cortical regions (dark blue). (*B*) Left PUTd connectivity. (*C*) Interaction between putamen subregion seed and target network (affective vs. motor). (*D*) Left PUTv connectivity after controlling for shared variance with left PUTd. Connection strengths in *A*, *B*, and *D* represent correlation t-statistics.

We found that PUTv in the left hemisphere exhibited significant functional connectivity with multiple motor [upper limb area of the motor cortex (M1_UL_), SMA, CMA] and affective (NBM, BLA, CeA) regions (summarized in the connectome plot in Fig. 2*A*; all *P* < 0.05, FDR-corrected). All connections were bilateral except for BLA, PMd, and M1_UL_, which were ipsilateral. PUTv was also functionally connected ipsilaterally with VL and GPi. A similar connectivity profile was observed with a PUTv seed in the right hemisphere (*SI Appendix*, Fig. S1). In contrast, PUTd was preferentially connected with motor regions (CMA, SMA, PMd, M1_UL_, PMv), showing connectivity with fewer affective regions (NBM, CeA) (Fig. 2*B*; all *P* < 0.05, FDR-corrected). NBM connections were bilateral, CeA was unilateral. Meanwhile, CMA and PMd were ipsilateral and remaining motor targets bilateral. PUTd was also functionally connected ipsilaterally with VL (left). A similar connectivity profile was observed with a PUTd seed in the right hemisphere (*SI Appendix*, Fig. S2).

To formally evaluate whether dorsal and PUTv subregions differed systematically in their functional connectivity with motor vs. affective networks, we tested for region-by-network differences using an ANOVA (Fig. 2*C*). This revealed a significant interaction between putamen subregion and target network [F(1,27) = 8.45, *P* = 0.007, η^2^ = 0.019]. Post hoc comparisons indicated that PUTd was selectively coupled to motor targets (*P* = 0.03, Bonferroni corrected, *d* = –0.72), whereas PUTv was functionally connected with both motor and affective regions to a similar degree, a profile potentially capable of linking the two. Thus, PUTd showed motor-selective connectivity, whereas PUTv showed both motor and affective connectivity.

Finally, to test whether PUTv connectivity (in particular, its coupling with motor regions) simply reflected shared variance with PUTd, we repeated the connectivity analysis after partialling out the ipsilateral PUTd time series. We replicated our results after imposing this additional constraint, whereby left PUTv remained functionally connected to key affective regions (BLA, CeA, NBM) as well as the motor-associated CMA (all *P* < 0.05; FDR-corrected; Fig. 2*D*), indicating integration with affective and, critically, motor circuits independently of PUTd. (Similar results were observed with this partial correlation method, using a PUTv seed in the right hemisphere; *SI Appendix*, Fig. S3). Two additional control analyses further clarified the specificity of these connectivity patterns. First, partialling out PUTv variance eliminated the PUTd–CMA association (*SI Appendix*, Figs. S4 and S5), suggesting that apparent PUTd–CMA connectivity is itself dependent on shared variance with PUTv. Second, the PUTv–CMA association remained robust when controlling for amygdala variance (*SI Appendix*, Figs. S6–S9), indicating that PUTv–CMA connectivity is independent of common amygdala inputs.

Collectively, these findings demonstrate that PUTv exhibits independent functional connectivity with both motor and affective networks, consistent with the proposed OLC architecture whereby PUTv provides a pathway through which affective and incentive signals influence motor output in healthy humans. A complementary model-free analysis using Functional Network Connectivity with hierarchical clustering corroborated these findings, with PUTv grouping with affective regions and showing significant connectivity to motor areas even after PUTd variance was removed (*SI Appendix*, Figs. S10 and S11).

### Experiment 2.

#### Task fMRI evidence that incentive conditions modulate movement initiation and circuit engagement.

**Fig. 3.**
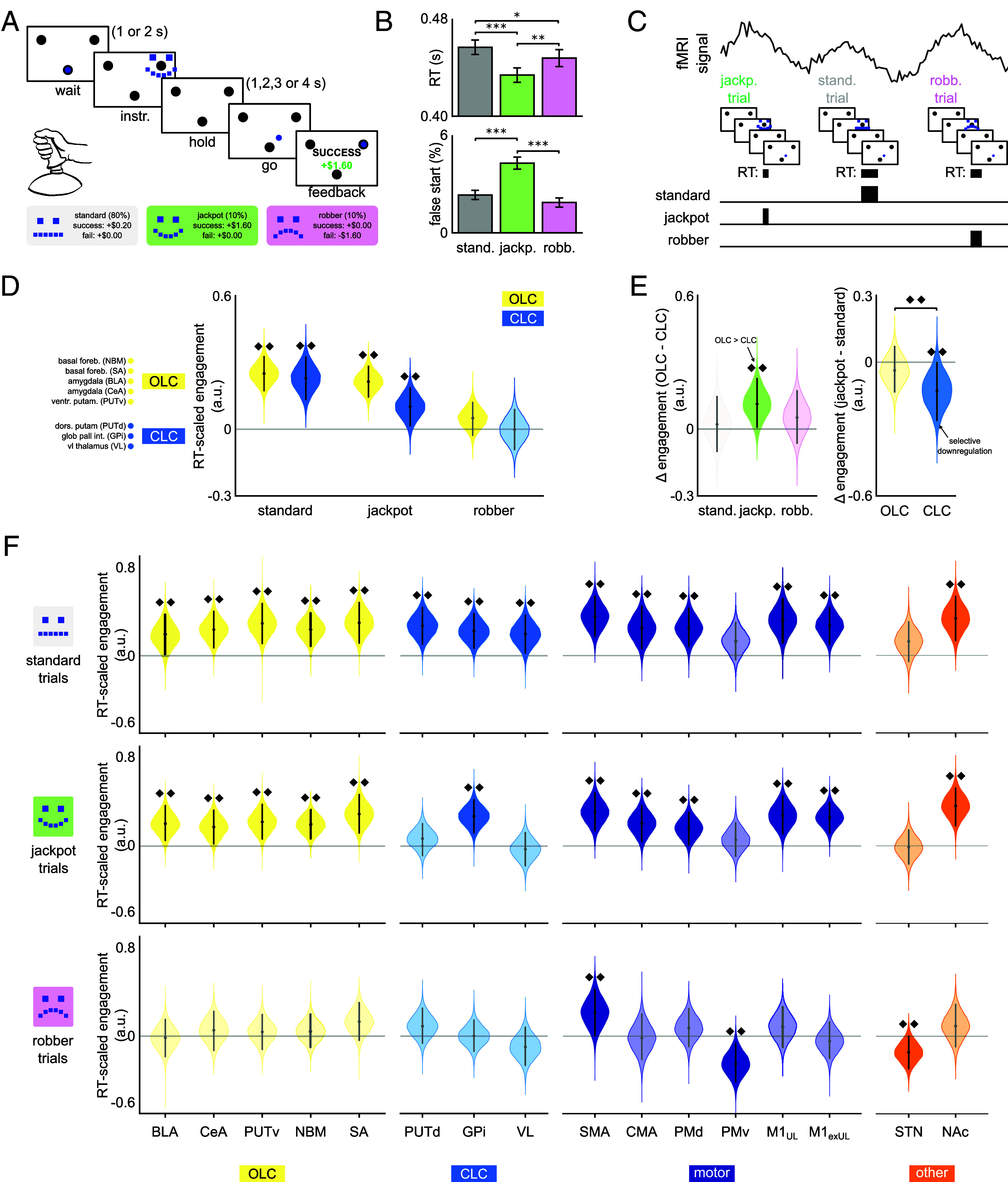
(*A*) Task design with jackpot, robber, and standard reward conditions. (*B*) Median movement initiation times (RT) and average false starts (movement prior to “go” cue) by incentive condition; error bars show SEM. (*C*) RT-scaled BOLD modeling approach. (*D*) Circuit-level engagement across conditions: both OLC and CLC engaged under standard reward, but neither under robber. (*E*) Relative circuit engagement across conditions and relative jackpot engagement across circuits, combine to demonstrate selective downregulation of CLC. (*F*) Node-level analysis for OLC, CLC, motor-cortical and other subcortical nodes across conditions. Violin plots (*D*–*F*) show Bayesian posterior distributions of the location parameter μ, where positive/negative values indicate greater BOLD response with shorter/longer RT. Dots indicate posterior mean, lines span HDI. *P < 0.05; ***P < 0.001; ♦♦ indicates a Bayesian credible effect: the posterior HDI for engagement excludes 0, or the HDI of the difference excludes 0 for pairwise comparisons.

To examine how varying incentive conditions modulate movement initiation and movement-related BOLD activity in CLC vs. OLC nodes, 68 healthy human participants (50 female, mean age 20.75 ± 1.86 y) performed a speeded precision joystick reaching task during an fMRI scan at 3 T. Each trial offered a jackpot ($1.60 high-reward), robber ($1.60 high-loss avoidance), or standard ($0.20 reward) incentive (Fig. 3*A*). Success was defined as the cursor reaching and being held at the cued target before a preset group deadline, which determined if incentives were met. Thus, the task requires both rapid initiation and precise online control for successful performance. The task comprised 300 trials in total (10% jackpot, 10% robber, 80% standard), with participants achieving 56.8% success overall (individual differences and temporal drift are shown in *SI Appendix*, Fig. S12 *A*, *B* and *F*).

We first assessed behavioral effects of incentive conditions using full-factorial repeated-measures ANOVAs including incentive condition, run, hold-period duration, and all interactions (all kinematic variables in *SI Appendix*, Fig. S13, Table S3; interaction drilldowns in *SI Appendix*, Fig. S14). Critically, incentives robustly modulated movement initiation speed [RT; Fig. 3*B*; F(1.8,122.0) = 18.5, *P* < 0.001], with both incentive conditions producing faster initiation than standard (jackpot 440 ms vs. standard 460 ms, *P*_fdr_ < 0.001; robber 453 ms vs. standard 460 ms, *P*_fdr_ = 0.027). Jackpot was additionally faster than robber (*P*_fdr_ = 0.002), establishing a graded facilitation that distinguished reward gain from loss avoidance. This RT facilitation was contextually stable: ordinal across hold-period durations (faster than standard at both short and long holds, both *P*_fdr_ < 0.001; *SI Appendix*, Fig. S14*A*) and did not significantly vary across runs (incentive × run, *P* = 0.056; *SI Appendix*, Table S3). 62 of 68 participants showed this jackpot RT advantage. Beyond initiation, the robber condition uniquely increased peak velocity [F(1.4,93.7) = 9.9, *P* < 0.001; *SI Appendix*, Figs. S13*D* and S15]. However, unlike the more stable RT facilitation, this effect interacted with hold duration and run in ways that were either disordinal or confined to early portions of the session (*SI Appendix*, Fig. S14 *B* and *F*). Strikingly, the jackpot condition uniquely increased false starts, that is, movements initiated prior to the go cue [Fig. 3*B*; F(1.4, 91.8) = 16.1, *P* < 0.001], and this was concentrated in Run 1 (*SI Appendix*, Fig. S14*E*). This pattern suggests the jackpot condition prioritized initiation speed at the cost of accuracy, particularly early in the experiment. The RT modulation by incentive was uncorrelated with individual differences in overall success rate or its change across the testing session (*SI Appendix*, Fig. S12 *C* and *G*).

#### RT-scaled BOLD activation reveals selective downregulation of CLC under positive incentive.

We next tested whether CLC and putative OLC nodes showed differential BOLD responses associated with movement initiation across incentive conditions. For this, we examined how BOLD signal duration in predefined CLC and OLC nodes (Fig. 1*C*) scaled with movement initiation speed. Analyses were restricted to 62 individuals showing jackpot-driven RT modulation. To contextualize these circuits within the motor system, we included motor cortical regions from Experiment 1 (SMA, CMA, PMd, PMv, M1_UL_) adding motor cortex excluding the upper limb region (M1_exUL_), as well as nucleus accumbens (NAc), given its ventral striatal proximity to PUTv, and subthalamic nucleus (STN), given its established role in movement inhibition (i.e., other in Figs. 1 and 3). We employed an extension of recent BOLD signal modeling approaches (33) using condition-specific RT-scaled regressors at movement initiation (*Materials and Methods*, Fig. 3*C*). Using a hierarchical Bayesian approach, we estimated RT-scaled BOLD activation at the group level for each node and condition ($\mu_{condition,region}$), which were then averaged across nodes at each posterior draw to derive circuit-level RT-scaled estimates ($\mu_{condition,circuit}$) (*Materials and Methods*, Fig. 3*D*). We reasoned that circuits facilitating movement initiation should show greater BOLD responses during faster movements. Accordingly, positive μ values indicate what we operationally define as engagement, i.e., greater BOLD response with faster movement initiation.

First, we examined OLC and CLC’s overall engagement in the three incentive conditions (Fig. 3*D*). In the standard condition, nodes of both circuits showed credible engagement, with posterior highest density intervals (HDIs) excluding zero in each case [$E(\mu_{standard,OLC})$ = 0.251, HDI = (0.175, 0.330); $E(\mu_{standard,CLC})$ = 0.229, HDI = (0.130, 0.322)]. This pattern replicated under jackpot [$E(\mu_{jackpot,OLC})$ = 0.215, HDI = (0.146, 0.284); $E(\mu_{jackpot,CLC})$ = 0.101, HDI = (0.013, 0.184)], albeit with weaker engagement in CLC (see below). In contrast, in the robber condition, neither circuit showed credible engagement, with HDIs including zero [$E(\mu_{robber,OLC})$ = 0.050, HDI = (−0.025, 0.122); $E(\mu_{robber,CLC})$ = −0.002, HDI = (−0.094, 0.089)]. Thus, nodes of both CLC and OLC are robustly engaged (greater BOLD response with faster RT) when movements are motivated by positive incentive (standard or jackpot). This engagement is notably absent in the robber condition, suggesting valence influences OLC and CLC involvement in movement initiation.

Next, we examined OLC and CLC’s relative engagement in the three incentive conditions, to probe for shifts in the balance of control (Fig. 3*E*). For this, we directly contrasted engagement across circuits (${\Delta_{\mathrm{condition}}=\mu }_{condition,OLC}-\mu_{condition,CLC}$) for each condition. In the standard condition, circuits showed no credible difference in engagement [${E(\Delta }_{\mathrm{standard}})$ = 0.021, 89% HDI = (−0.102, 0.144)], suggesting balanced engagement during movement motivated by standard reward. Similarly, under robber, no credible difference emerged between circuits [${E(\Delta }_{\mathrm{robber}})$ = 0.052, HDI = (−0.059, 0.175)], though taken with results above, this likely reflects mutual disengagement rather than balanced activation.

Strikingly, in the jackpot condition, OLC nodes showed credibly stronger engagement than CLC [${E(\Delta }_{\mathrm{jackpot}})$ = 0.113, HDI = (0.004, 0.224)]. To directly test whether this constitutes selective downregulation of CLC, independent of dynamics in OLC, we examined within-circuit changes from standard to jackpot conditions (${\Delta_{\mathrm{circuit}}=\mu }_{jackpot,circuit}-\mu_{standard,circuit}$) for each circuit (Fig. 3*E*). This revealed that while OLC nodes maintained consistent engagement across both conditions [${E(\Delta }_{\mathrm{OLC}})$ = −0.036, HDI = (−0.141, 0.068)], CLC showed credible downregulation under jackpot [${E(\Delta }_{\mathrm{CLC}})$ = −0.128, HDI = (−0.256, −0.002)].

To pinpoint the sources of the apparent CLC downregulation, we next examined engagement at the level of individual nodes (Fig. 3*F*). Consistent with stable engagement, all OLC nodes were credibly engaged in both standard and jackpot conditions (0 ∉ all posterior HDIs; Fig. 3 *F*, “OLC” panels, *Upper* and *Middle* row). In contrast, within the CLC (“CLC” panels), a selective shift emerged: all nodes were engaged in the standard condition (0 ∉ all posterior HDIs; Fig. 3 *F*, *Upper* row), but under jackpot, only GPi remained engaged [${E(\mu }_{GPi,jackpot})$ = 0.268, HDI = (0.118, 0.418); Fig. 3 *F*, *Middle* row]. Both VL and PUTd no longer showed credible engagement (0 ∈ each posterior HDI; [${E(\mu }_{VL,jackpot})$ = −0.030, HDI = (−0.180, 0.122)]; ${E(\mu }_{PUTd,jackpot})$ = 0.066, HDI = (−0.087, 0.201); Fig. 3 *F*, *Middle* row}, implicating their downregulation as primary contributors to reduced CLC engagement in the jackpot condition.

The node-level results (Fig. 3*F*) further reveal that the jackpot-driven engagement shifts (relative to standard) were subcortically selective. Beyond our a priori CLC and OLC nodes, we included motor-cortical regions, in addition to NAc and STN in our hierarchical Bayesian model to contextualize circuit-level effects within the broader motor system. Engagement patterns for these regions are respectively shown in the “motor” and other panels of Fig. 3*F*. A striking dissociation emerged: whereas CLC nodes (PUTd, VL) showed reduced engagement from standard to jackpot, all motor-cortical regions (SMA, CMA, PMd, PMv, M1_UL_, M1_exUL_) maintained stable engagement in both conditions. With the exception of PMv, all motor-cortical regions showed credible engagement under both standard and jackpot. In addition, NAc showed credible engagement in both standard and jackpot conditions, whereas STN did not show credible engagement in either. This pattern indicates that jackpot-driven downregulation specifically targets nodes in the CLC rather than reflecting a general motor or reward system reconfiguration.

Even more strikingly, the robber condition elicited a dramatic cortical reconfiguration. Unlike the stability observed across positive-incentive conditions (i.e., standard and jackpot), most motor-cortical nodes did not show credible engagement under robber (Fig. 3 *F*, motor panel, *Bottom* row), mirroring the subcortical disengagement observed in this condition (i.e., OLC and CLC in Fig. 3 *F*, *Bottom* row). SMA emerged as the sole region (cortical or subcortical) maintaining credible engagement across all three incentive conditions, suggesting a condition-invariant role in movement initiation. Remarkably, PMv (motor) and STN (other), neither of which were credibly engaged under standard or jackpot, uniquely exhibited negative engagement under robber. Negative engagement reflects greater BOLD response for slower RTs, i.e., the opposite pattern from nodes engaged in the two reward conditions. This inverse, valence-specific recruitment of PMv and STN may therefore implicate the robber condition (i.e., loss-avoidance) in uniquely engaging stopping (i.e., inhibitory or hesitation) processes that remain dormant during the reward-motivated movement in the other two conditions.

Collectively, these findings reveal incentive-specific engagement of CLC, OLC, and motor-cortical areas during movement initiation. In the standard condition, both OLC and CLC showed robust engagement (greater BOLD response with faster RT), reflecting coordinated subcortical contributions to movement initiation. High-reward (jackpot) incentive selectively downregulated CLC (particularly PUTd and VL) while preserving OLC engagement, with this subcortical reconfiguration occurring against stable motor-cortical engagement. By contrast, loss-avoidance (robber) incentive eliminated credible engagement in both subcortical circuits, reduced engagement across most motor-cortical regions (excepting SMA), and produced negative engagement (greater BOLD response with slower RT) in regions associated with stopping (PMv and STN). These divergent patterns point to a versatile architecture for movement initiation that flexibly engages distinct cortico-subcortical circuits depending on both the salience and valence of incentive conditions.

## Discussion

Affective signals influence motor control, potentially by way of the brain maintaining multiple parallel cortico-subcortical circuits differentially engaged by motivational context. Neuroanatomical evidence in NHPs and clinical observations in humans suggest a PUTv-centered circuit with connectivity to affective systems (OLC) may operate alongside the canonical sensorimotor circuit through PUTd (CLC), but whether these circuits exist as functionally independent in humans and, critically, whether they are differentially recruited under varying incentive conditions remains unknown. We therefore conducted two complementary neuroimaging experiments.

We first report connectomic data acquired using ultra-high-field multi-echo fMRI, enabling precise regional delineation and mitigation against signal dropout, particularly in the basal ganglia (34). These data reveal that PUTv maintains balanced connectivity with both affective and motor networks, while PUTd couples preferentially to motor targets. Critically, dual connectivity persisted in PUTv even after controlling for shared variance with PUTd, suggesting functional independence from the traditional motor circuit.

Within this partialled map, PUTv showed prominent connections with amygdala nuclei [central (CeA) and basolateral (BLA)], consistent with NHP tracer evidence identifying amygdala structures as inputs to PUTv (7, 8, 35). These findings complement existing observations of a dorsal/ventral striatum gradient, whereby ventral striatum is characteristically closely associated with subcortical areas relevant for affective and motivational processes (17, 36–38). We additionally observed robust associations between PUTv and basal forebrain (NBM). While this structure may offer cholinergic input to PUTv, NHP evidence also supports its role as an output target. NHP ventral striatum (combining PUTv and NAc) bypasses the canonical GPi route to the motor cortex (35, 39), targeting instead ventral pallidum and NBM, whose cholinergic outputs widely target the cerebral cortex (40–42).

PUTv signal also covaried with a broad suite of motor and premotor areas, however, most of these connections were eliminated when PUTd variance was removed, suggesting they likely reflected striato-motor connectivity mediated by PUTd. Nevertheless, PUTv robustly covaried with CMA, situated on the inferior medial wall of the caudal frontal lobe. This association persisted when controlling for both PUTd and amygdala variance, positioning CMA as a candidate node through which PUTv may influence motor circuits. This finding is noteworthy because CMA’s functional properties align with postselection affective modulation of motor function. CMA is classically considered a specialized hub for translating affective drive into action (43), and preferentially activates for self-initiated movements (44). Recent network-neuroscience evidence further suggests that it sits downstream of decision-related regions where it initiates and implements selected actions (45). The inputs to CMA further support this postselection modulatory role: both PUTv and the BLA encode conjunctions of incentive valence and action tendency (46–48), but do not represent detailed movement parameters. Critically, CMA’s projections to premotor spinal laminae favor global action initiation rather than fine motor control (49–51), distinguishing it from lateral motor areas involved in precise movement execution. While CLC and OLC configurations involving CMA and basal ganglia remain to be characterized in primates, these converging functional and anatomical properties position PUTv’s preserved affective connectivity and association with CMA as an alternative route through which affective signals may influence movement initiation beyond canonical sensorimotor circuits. Direct demonstration of this functional pathway awaits further investigation.

We next report behavioral and task-derived BOLD data to examine how incentive conditions modulate initiation speed and the engagement profile of OLC nodes relative to those in CLC. First, at the behavioral level, we revealed a clear asymmetry in incentive effects: high reward (jackpot) selectively expedited movement initiation relative to both standard and high-loss avoidance (robber) conditions. Concurrently, participants also showed increased false starts under jackpot, consistent with participants optimizing for speed at the cost of accuracy under high positive incentive.

Task-derived BOLD activation was then assessed to determine which engagement pattern best explained circuit dynamics across incentive conditions: additive, competitive, or selective downregulation. Modeling RT-scaled BOLD activations allowed us to infer whether circuit nodes operationally scaled their activity with initiation speed. Under standard reward conditions, OLC, CLC, and cortical-motor nodes showed comparable RT-scaled activation, suggesting balanced engagement across the brain during typical goal-directed movement. However, incentive salience and valence drove divergent patterns of engagement, with robber and jackpot conditions seemingly engaging qualitatively distinct control architectures.

The high-reward (jackpot) incentive produced a striking dissociation consistent with our selective downregulation hypothesis: OLC nodes maintained engagement (relative to the standard condition) while CLC nodes disengaged. Node-level analysis revealed this downregulation was subcortically selective, driven by reduced engagement in PUTd and VL thalamus, while motor cortical regions maintained stable engagement (CMA, SMA, PMd, M1). Notably, NAc exhibited an engagement profile indistinguishable from PUTv, despite likely receiving distinct amygdala inputs (16). Behaviorally, jackpot drove faster initiation alongside more false starts, a clear speed–accuracy trade-off. This pattern suggests that high positive incentive downweights deliberative sensorimotor basal ganglia circuits, resulting in leaner corticostriatal circuitry predominantly involving PUTv and affective regions, while prioritizing speed over accuracy, at least in early trials (26–28). Critically, CMA maintained engagement under jackpot. As discussed above, CMA’s properties favor global movement initiation over fine control. In addition, Experiment 1 established robust functional connectivity between CMA and PUTv, with basal forebrain as a plausible relay. Thus, one parsimonious interpretation is that with reduced CLC influence, affective signaling from PUTv (and possibly NAc) through CMA may gain relative modulatory prominence in shaping rapid motor output.

By contrast, the high-loss avoidance (robber) incentive revealed a pattern consistent with our competitive shift hypothesis. In the robber condition, neither OLC nor CLC showed credible RT-scaled activation, indicating disengagement of these circuits. Instead, robber uniquely recruited PMv and STN, regions that remained dormant under both standard and jackpot. Moreover, these regions exhibited negative engagement: greater activation with slower RT, suggesting recruitment of hesitation or stopping processes that compete with movement initiation. This pattern, i.e., distinct regions with different behavioral correlates, is consistent with broader evidence from human learning and decision-making, where rewards and costs recruit qualitatively distinct mechanisms rather than encoding valence through opposing responses in shared circuits (52, 53). The negative engagement in PMv is also consistent with recent evidence in PD patients, where increased cautiousness with movement appears to shift control away from striatum to cortical areas (54). Thus, high-loss avoidance appears to engage stopping processes that compete with and may even supplant the circuits supporting movement initiation under positive incentive conditions.

**Fig. 4.**
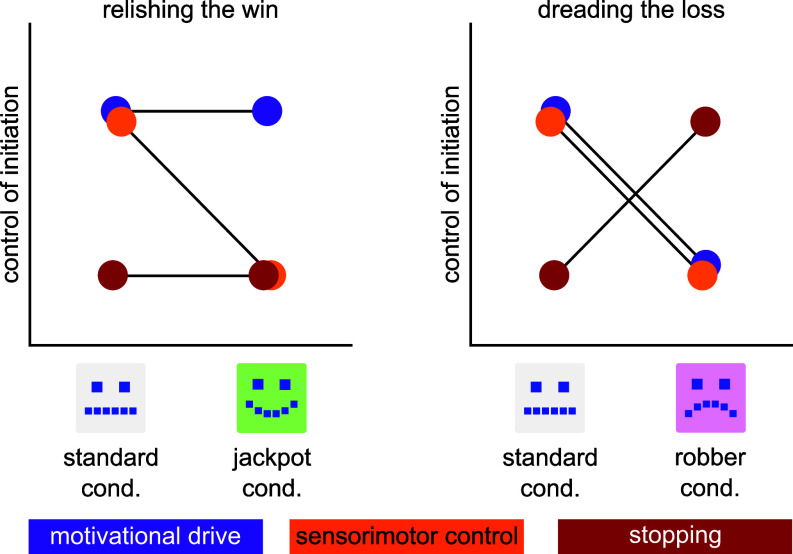
Proposed multilevel model whereby phenomenology (i.e., the subjective construal of incentive as opportunity vs. threat) determines which neural architecture dominates movement initiation

The differential engagement patterns across incentive conditions are consistent with a multilevel control architecture, proposed in Fig. 4. Traditional action-selection models posit that competing action plans (including stopping or hesitation) vie for motor output (55). Our findings extend this framework, suggesting that phenomenology evoked by incentive conditions determines which of several coexisting control architectures dominates. Despite jackpot and robber conditions being economically equivalent, they elicited entirely distinct behavior and neural configurations. This suggests the controlling architecture itself may reconfigure based on phenomenology, specifically, the asymmetric weighting of incurred losses vs. forgone gains. This would constitute a flexible control that accommodates when the greatest cost is hesitation, and also when the greatest cost is haste.

What is notable is that this reconfiguration emerged in the kinematic structure of the action itself. Our task adopted the valence and salience design of the MID but required speeded precision reaches rather than button presses. In the button-press MID literature, behavioral measures predominantly do not differentiate reward from loss anticipation (56–59), with the few exceptions disagreeing on direction (60–62). At the neural level, meta-analytic evidence suggests valence-specific activation is largely deferred to the outcome phase, i.e., after feedback on whether incentives were met (32). Our results place neural reconfiguration during movement onset, before outcomes are known. Thus, a task with greater motor demands may provide the behavioral resolution to create more dimensions along which conditions can differentiate performance. Indeed, reaching tasks can separate reward and punishment effects, at least at the behavioral level (63). Yet this facilitation can reverse when phenomenology shifts: large promised rewards degrade skilled motor performance, consistent with a spontaneous reframing of anticipated gain as potential loss (64). Separable control across reward and loss conditions is thus unlikely to be a fixed property of incentive valence alone, but rather depends on the interaction between phenomenological context and the motor ecology through which goals are attained.

The architectural reconfiguration described above was observed in the 62 of 68 participants who showed jackpot-driven RT facilitation; neuroimaging analyses were restricted to this subsample to ensure a consistent behavioral signal for interpretable BOLD contrasts. That said, initiation speed is one kinematic channel among several available in a precision reaching task. Execution velocity, for instance, carried an incentive signal in our data but did not do so stably across task contexts. Indexing neural engagement by a different kinematic channel, or under task contexts that more stably recruit it, may reveal different architectural configurations entirely.

Our present findings necessarily remain correlational. In the connectivity findings in particular, we cannot definitively establish that the affective regions, PUTv and CMA, form a directional circuit. Indeed, the coactivation patterns we observe could reflect common upstream drivers, feedback loops, or other noncausal relationships. Nevertheless, within the constraints of human neuroimaging, we advance the evidence base in specific ways. First, our ultra-high-field multi-echo acquisition, signal decomposition, and partialling out of PUTd variance provide strong resolution for establishing a functional integration for PUTv that is independent of nodes in canonical sensorimotor circuits. Second, we employ RT-scaled BOLD modeling that identifies which circuit nodes operationally scale their activity with initiation speed under different incentive conditions.

An important step toward greater causal inference is to translate our findings into clinical experiments, particularly in PD where convergent evidence suggests therapeutic potential. As we noted, PUTv is relatively spared in both NHP PD models (21, 22) and in humans with PD, at least early in the disease. Amygdala–PUTv connectivity likewise remains preserved, at least until mid-stage disease progression (65). Moreover, the disease shows divergent trajectories across motor circuits: decreased SMA-putamen connectivity yet preserved CMA function (66, 67). Together, these findings suggest that key nodes of the proposed OLC pathway remain anatomically viable even as the canonical CLC degrades. Functionally, ventral striatal incentive responses in PD are not absent but temporally displaced, emerging at the salient event itself rather than in anticipation of it (68). Our model suggests that paradoxical kinesia (PK) in some PD patients may occur when affectively salient contexts engage this preserved pathway, shifting control from impaired deliberative circuits. However, striatal-bypass accounts, via brainstem or cerebellum, additionally offer plausible alternative models for PK (69). A greater understanding of how the brain flexibly supports voluntary movement across affectively salient contexts may open new therapeutic avenues, from lifestyle interventions that leverage such contexts to refined deep-brain stimulation protocols.

## Materials and Methods

### Experiment 1.

#### Participants and MRI acquisition.

28 healthy, right-handed participants (15 M/13F, ages 21 to 39) were scanned at 7 T (Siemens Terra) with a 32-channel receive head coil. The protocol included T1-weighted MPRAGE anatomical imaging (0.75 mm^3^), multi-echo resting-state fMRI (2 × 2 × 1.5 mm, 10:52 min), and spin-echo field maps for distortion correction. Physiological signals (respiration, pulse) were recorded during scanning. All participants provided written informed consent for study procedures approved by the Institutional Review Boards at University of California, Santa Barbara, and the University of Southern California. Full acquisition parameters and participant screening criteria are provided in *SI Appendix*, Methods S1.

#### fMRI preprocessing.

Multi-echo fMRI data were preprocessed using ANTs, FSL, and Tedana (v24.0.1). Preprocessing included motion correction, slice-time correction, fieldmap-based distortion correction (available for 16/28 participants), and coregistration to T1 anatomy. Tedana was used to optimally combine echoes and remove non-BOLD signal components via PCA/ICA. Denoised data were normalized to MNI-152 space for connectivity analysis. Full preprocessing details are provided in *SI Appendix*, Methods S2.

#### Functional connectivity analysis.

Functional connectivity was computed using the CONN toolbox (v22.a). Additional confound regression included CompCor-derived anatomical noise components and bandpass filtering (0.008 to 0.09 Hz). Seed-to-voxel connectivity was estimated via bivariate correlation, with significance assessed using FDR correction (α = 0.05). To isolate PUTv-specific connectivity, partial correlations were computed after regressing the ipsilateral PUTd time series. Network-level analyses compared connectivity to motor (CMA, SMA, PMd, M1_UL_, PMv) and affective (BLA, CeA, NBM) regions-of-interest (ROIs) using repeated-measures ANOVA. Full details are provided in *SI Appendix*, Methods S3.

### Experiment 2.

#### Participants and MRI acquisition.

68 healthy participants (50F/18 M, mean age = 20.75 y; SD = 1.86) were scanned at 3 T (Siemens Prisma) with a 64-channel head/neck coil. The protocol included T1-weighted MPRAGE anatomical imaging (0.94 mm^3^), gradient-echo field maps for distortion correction, and task fMRI (2.5 mm^3^, TR = 1,900 ms) acquired during three runs. All participants provided written informed consent for study procedures approved by the Institutional Review Board at University of California, Santa Barbara. Full acquisition parameters and participant screening criteria are provided in *SI Appendix*, Methods S4.

#### Incentivized vigor task.

Participants performed a speeded precision joystick reaching task under three incentive conditions: jackpot ($1.60 reward), robber ($1.60 loss avoidance), or standard ($0.20 reward). Joystick position was mapped to screen cursor position, with full deflection calibrated to the screen edge and targets positioned within this range, allowing overshoots. The task comprised 300 trials (10% jackpot, 10% robber, 80% standard) across three runs. Success required reaching the cued target within a fixed deadline (1.87 s), calibrated from pilot data to yield ~50% success. Behavioral analyses used repeated-measures ANOVA with factors of incentive condition, hold period, and run, and all interactions. Full task details are provided in *SI Appendix*, Methods S5.

#### fMRI preprocessing and modeling.

Echo-planar imaging (EPI) data were preprocessed using ANTs and FSL, including motion correction, slice-time correction, fieldmap-based distortion correction, and normalization to MNI-152 space with 5 mm smoothing. First-level GLMs included instruction-phase regressors (stick functions at cue onset) and go-phase regressors (duration-scaled by RT) for each incentive condition, plus nuisance regressors for hold period and reach execution; the latter spans from movement initiation to trial end, reducing contamination of the RT-scaled estimate by outcome-related activity. ROI-averaged parameter estimates were submitted to hierarchical Bayesian models with Student’s t likelihoods to accommodate outliers. Credible activation was determined by 89% highest-density intervals excluding zero. Full preprocessing, modeling, and inference details are provided in *SI Appendix*, Methods S6.

## Supplementary Material

Appendix 01 (PDF)

## Data Availability

Anonymized Behavior and Neural data have been deposited in Zenodo (https://doi.org/10.5281/zenodo.19243767) (70). All other data are included in the manuscript and/or *SI Appendix*.
