# Supplementary material for: Incentive valence differentially engages open- and closed-loop basal ganglia circuits during movement initiation
Source: Proc Natl Acad Sci U S A. 2026 May 6;123(19):e2537314123. doi: 10.1073/pnas.2537314123 (PMC13167725; doi:10.1073/pnas.2537314123)
Supplement: Supplementary file 1 — Appendix 01 (PDF) [file pnas.2537314123.sapp.pdf]

Supporting Information for Incentive valence differentially engages open- and closed-loop basal ganglia circuits during movement initiation

Note: Clickable navigation links are provided in the footer of each page.

Table S1 Glossary of abbreviations

Table S2 Regions of interest: atlas sources and definitions

Table S3 Main behavior effects from repeated measures ANOVA

Figure S1 Connectome plot; seed in right PUTv

Figure S2 Connectome plot; seed in right PUTd

Figure S3 Connectome plot; seed in right PUTv (partial correlation\PUTd)

Figure S4 Connectome plot; seed in left PUTd (partial correlation\PUTv)

Figure S5 Connectome plot; seed in right PUTd (partial correlation\PUTv)

Figure S6 Connectome plot; seed in left PUTv (partial correlation\BLA)

Figure S7 Connectome plot; seed in right PUTv (partial correlation\BLA)

Figure S8 Connectome plot; seed in left PUTv (partial correlation\CeA)

Figure S9 Connectome plot; seed in right PUTv (partial correlation\CeA)

Figure S10 Connectome plot; model free analysis of all left-hemisphere ROIs

Figure S11 Connectome plot; model free analysis of all left-hemisphere ROIs (partial correlation\PUTd)

Figure S12 Task success variance across participants and task

Figure S13 Kinematic variables for each incentive condition

Figure S14 Interaction drilldowns for incentive effects modulated by hold-period duration or run

Figure S15 Velocity traces by incentive condition

SI Methods S1 Experiment 1: Participants and Study Protocol

SI Methods S2 Experiment 1: Multi-Echo fMRI Preprocessing and Denoising with Tedana

SI Methods S3 Experiment 1: CONN Functional Connectivity Analysis

SI Methods S4 Experiment 2: Participants and Study Protocol

SI Methods S5 Experiment 2: Incentivized Vigor Task

SI Methods S6 Experiment 2: Task-Based fMRI Preprocessing, Modeling, and Bayesian Inference

SI References Shared bibliography for SI Methods

Table S1 Glossary of abbreviations.

Abbreviation	Definition
BLA	Basolateral amygdala
BOLD	Blood-oxygen-level-dependent
CeA	Central nucleus of the amygdala
CLC	Closed-loop circuit
CMA	Cingulate motor area
GPi	Globus pallidus internus
EPI	Echo planar imaging
HDI	89% highest density interval
fMRI	Functional magnetic resonance imaging
$M1_{UL}$	Primary motor cortex, upper-limb region
$M1_{exUL}$	Primary motor cortex, excluding upper-limb region
NAc	Nucleus accumbens
NBM	Nucleus basalis of Meynert
NHP	Nonhuman primates
OLC	Open-loop circuit (putative)
PD	Parkinson's disease
PET	Positron emission tomography
PK	Paradoxical kinesia
PMd	Dorsal premotor cortex
PMv	Ventral premotor cortex
PUTd	Dorsal (sensorimotor) putamen
PUTv	Ventral (limbic) putamen
ROI	Region of interest
RT	Reaction time
SA	Septal area of the basal forebrain
SMA	Supplementary motor area
SNpc	Substantia nigra pars compacta
STN	Subthalamic nucleus
VL	Ventrolateral thalamus

Table S2 Regions of interest: atlas sources and definitions

ROI	Atlas	Description	Reference
Open-Loop Circuit			
BLA	Tyszka amygdala	BL masks combined (BL_BLV, BLN_BL_BLD+BLI, BLN_BM, BLN-La)	[1]
CeA	Tyszka amygdala	Central nucleus masks (CEN + CMN)	[1]
PUTv	Harvard-Oxford	Putamen ≥50%, voxels below MNI z = -1	[2]
NBM	JuBrain (SPM)	Sublenticular basal forebrain (Ch4)	[3]
SA	JuBrain (SPM)	Septum & horizontal diagonal band (Ch1-3)	[3]
Closed-Loop Circuit			
PUTd	Harvard-Oxford	Putamen ≥50%, voxels above MNI z = -1	[2]
VL	Julich Brain	VLA + VLP, 207-area atlas ≥50%	[4]
GPi	Tyszka subcortical	Deterministic atlas region	[5]
Motor & Premotor Cortex			
$M1_{UL}$	Brainnetome	Upper limb M1 ≥50%	[6]
$M1_{exUL}$	Brainnetome	Face, Trunk, Tongue M1 ≥50%	[6]
CMA	Hand-drawn	STG-defined using MNI coords, SMA reference	[7]
SMA	Julich Brain	Area 6mp, 207-area atlas ≥50%	[4]
PMd	Julich Brain	Areas 6d1-3, 207-area atlas ≥50%	[4]
PMv	Julich Brain	Areas 6r1 + 6v1-3, 207-area atlas ≥50%	[4]
Other			
NAc	Harvard-Oxford	Accumbens ≥50%	[8]
STN	Tyszka subcortical	Deterministic atlas region	[5]

Note: All ROIs defined in MNI152 standard space. PUTv/PUTd threshold based on Talairach z = 2 converted to MNI z = -1.

Table S3 Repeated Measures ANOVA: Main Effects and Two-Way Interactions

		Incentive			Run			Hold			Incentive×Hold			Incentive×Run			Run×Hold		
Variable	Stat	*F*	*df*	*p*	*F*	*df*	*p*	*F*	*df*	*p*	*F*	*df*	*p*	*F*	*df*	*p*	*F*	*df*	*p*
success rate	Mean	3.2	1.7,115.5	0.052	61.1	1.9,129.0	**<.001**	23.6	1,67	**<.001**	4.4	1.8,122.6	**0.017**	1.9	3.2,216.3	0.121	0.0	1.9,126.9	0.977
RT	Median	18.5	1.8,122.0	**<.001**	6.1	1.8,118.2	**0.004**	110.5	1,67	**<.001**	6.6	1.7,116.3	**0.003**	2.6	2.9,196.2	0.056	7.4	1.8,119.3	**0.001**
false starts	Mean	16.1	1.4,91.8	**<.001**	37.1	2.0,131.1	**<.001**	0.2	1,67	0.668	2.9	1.7,112.5	0.068	8.0	2.7,178.6	**<.001**	2.0	1.9,124.8	0.137
max. velocity	Median	9.9	1.4,93.7	**<.001**	0.5	1.6,105.4	0.541	4.5	1,67	**0.037**	4.7	1.5,101.2	**0.019**	4.7	2.7,182.9	**0.005**	0.1	1.7,114.2	0.832
max. acceleration	Median	18.0	1.4,93.3	**<.001**	1.1	1.8,117.7	0.318	0.2	1,67	0.645	2.2	1.5,102.9	0.126	2.8	2.5,164.3	0.051	2.5	1.9,124.9	0.091
time to max. vel.	Median	15.9	1.8,118.4	**<.001**	7.8	1.8,122.6	**<.001**	97.7	1,67	**<.001**	2.3	1.8,121.6	0.110	3.2	2.8,190.1	**0.026**	6.7	1.8,121.6	**0.002**
time to max. acc.	Median	5.0	1.7,116.5	**0.011**	8.9	1.9,128.7	**<.001**	61.3	1,67	**<.001**	5.3	1.9,127.1	**0.007**	2.3	3.3,218.7	0.069	5.3	1.9,124.8	**0.007**
initial X position	Median	1.6	1.7,116.2	0.204	0.9	1.8,121.4	0.409	0.1	1,67	0.822	1.6	1.6,105.8	0.204	0.7	3.1,210.4	0.576	1.4	2.0,131.9	0.240
initial Y position	Median	0.2	1.8,123.7	0.794	5.1	1.8,122.0	**0.009**	1.8	1,67	0.183	2.4	1.8,122.6	0.101	0.7	3.0,200.8	0.570	0.0	1.9,126.0	0.961
time to quarter acc.	Median	18.6	1.8,119.9	**<.001**	8.1	1.8,118.5	**<.001**	111.6	1,67	**<.001**	4.3	1.7,115.3	**0.021**	3.0	2.8,188.8	**0.035**	6.0	1.8,118.7	**0.005**

Note: Bold = $p < 0.05$. *df* = numerator, denominator. Greenhouse-Geisser correction applied for effects with numerator $df > 1$ (corrected *df* shown as decimals).

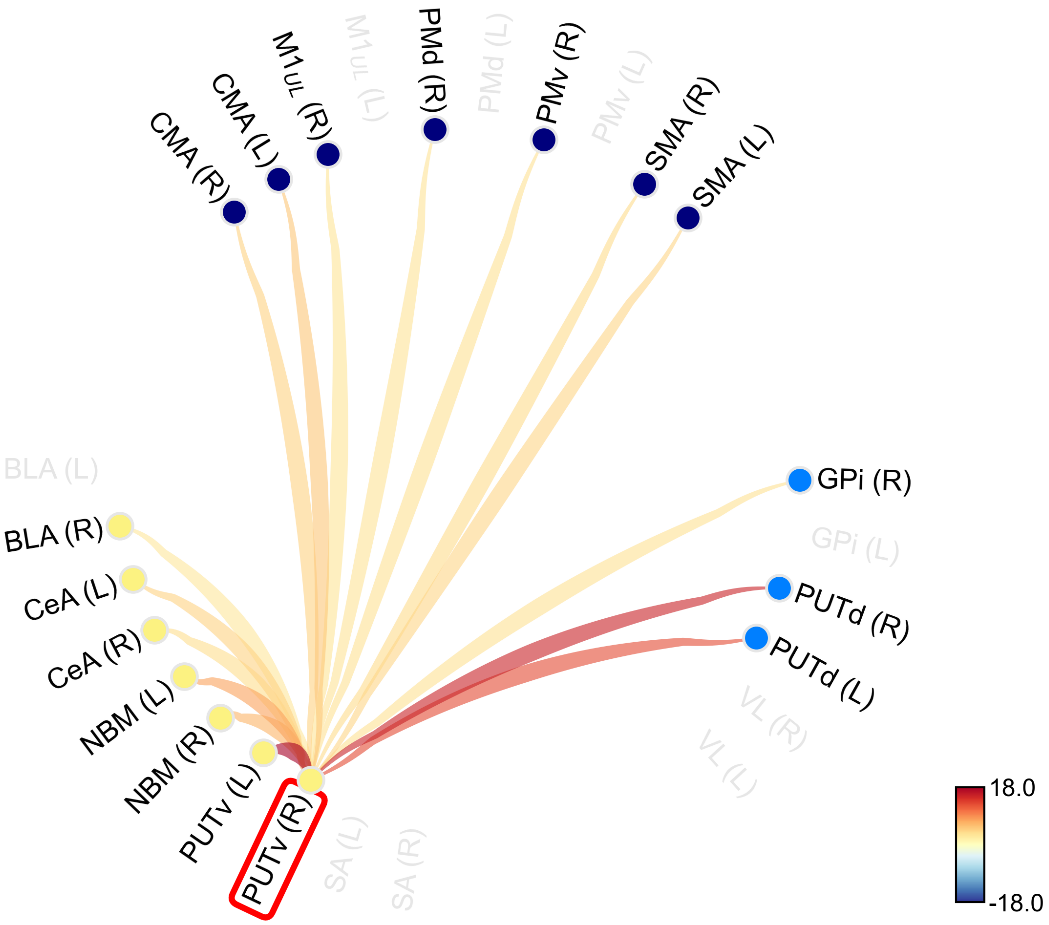

Figure S1 Connectome plot using seed in right ventral putamen

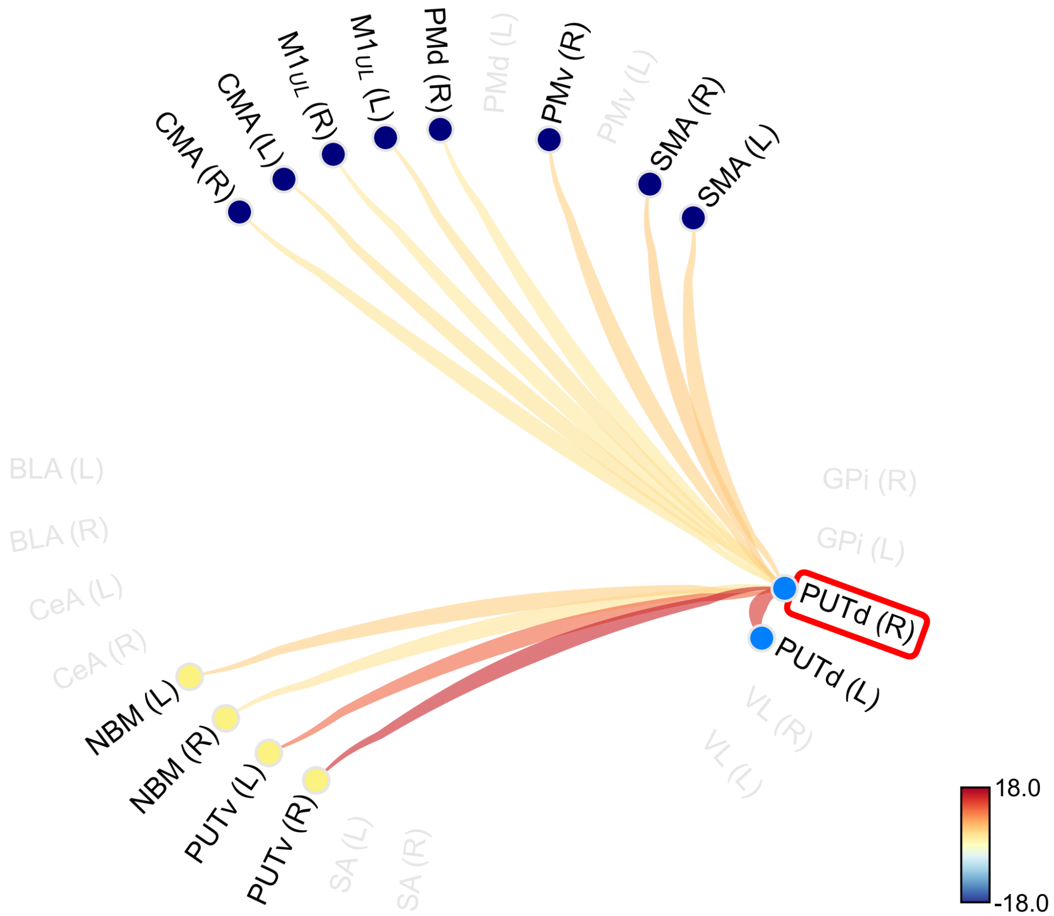

Figure S2 Connectome plot using seed in right dorsal putamen

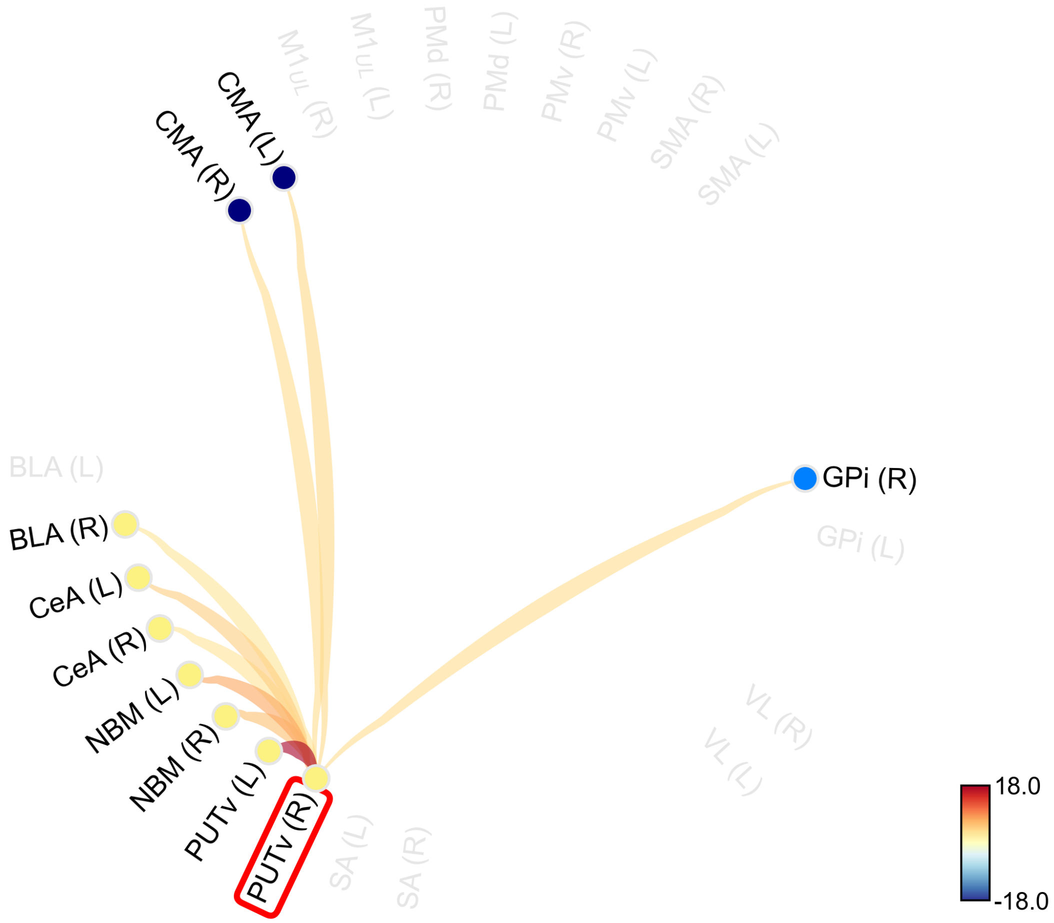

Figure S3 Connectome plot using seed in right ventral putamen; partial correlation (variance from dorsal putamen removed)

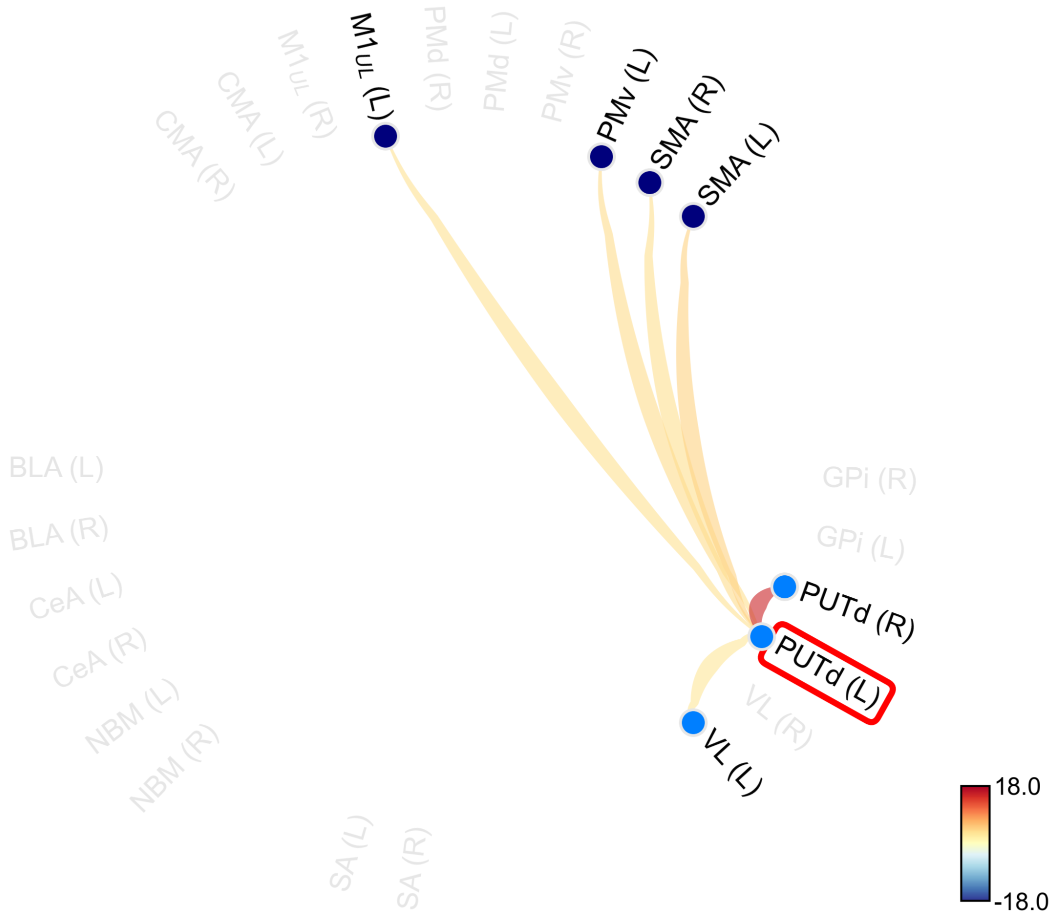

Figure S4 Connectome plot using seed in left dorsal putamen; partial correlation (variance from ventral putamen removed)

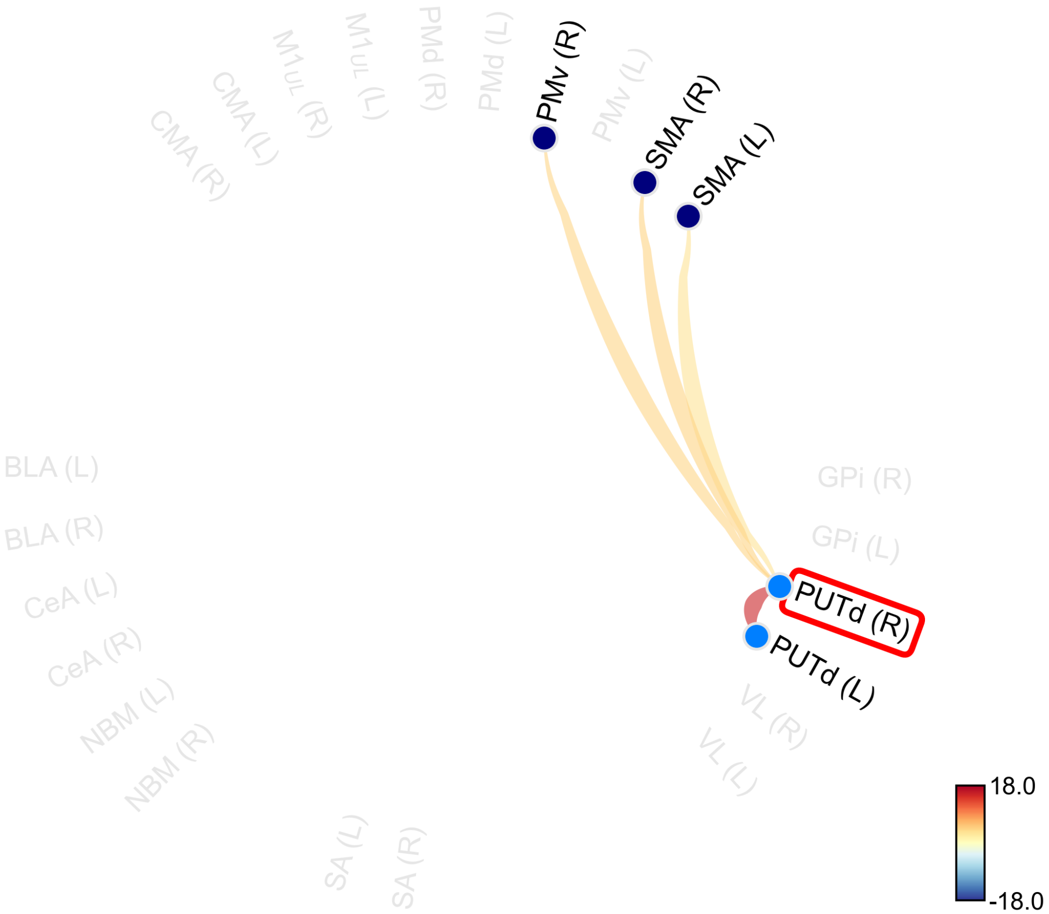

Figure S5 Connectome plot using seed in right dorsal putamen; partial correlation (variance from ventral putamen removed)

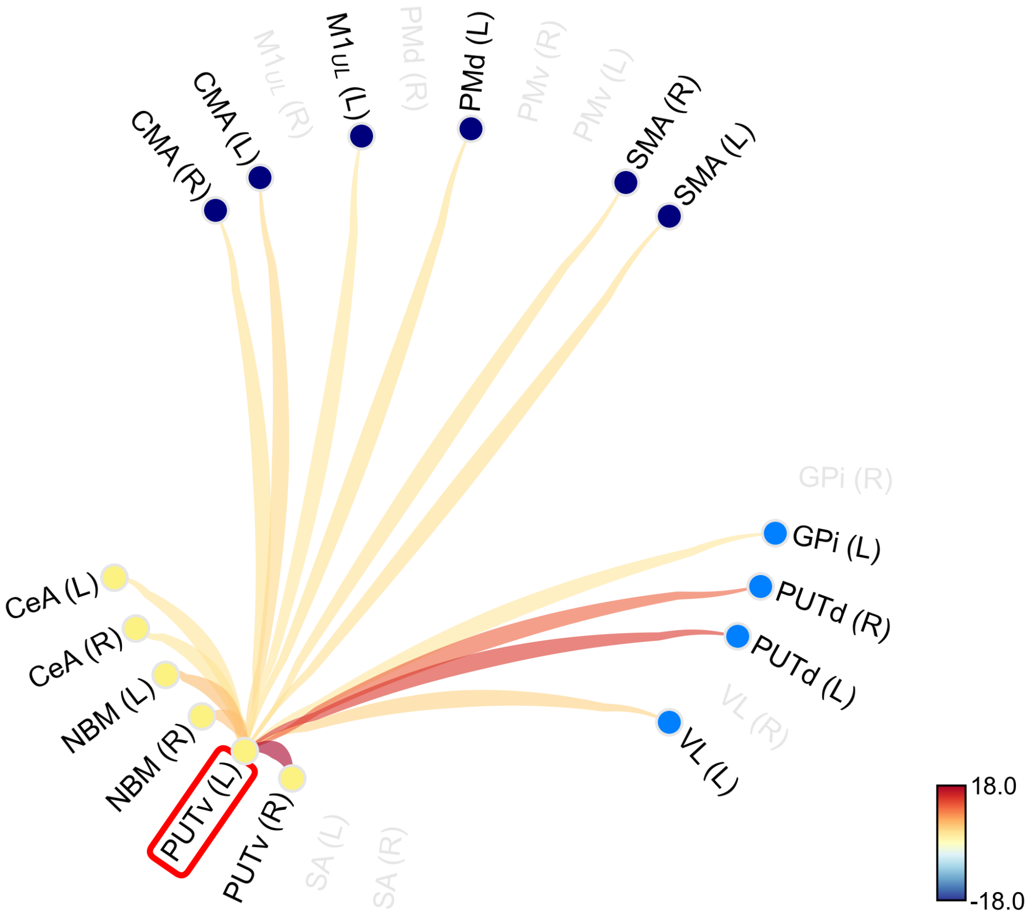

Figure S6 Connectome plot using seed in left ventral putamen; partial correlation (variance from basolateral amygdala removed)

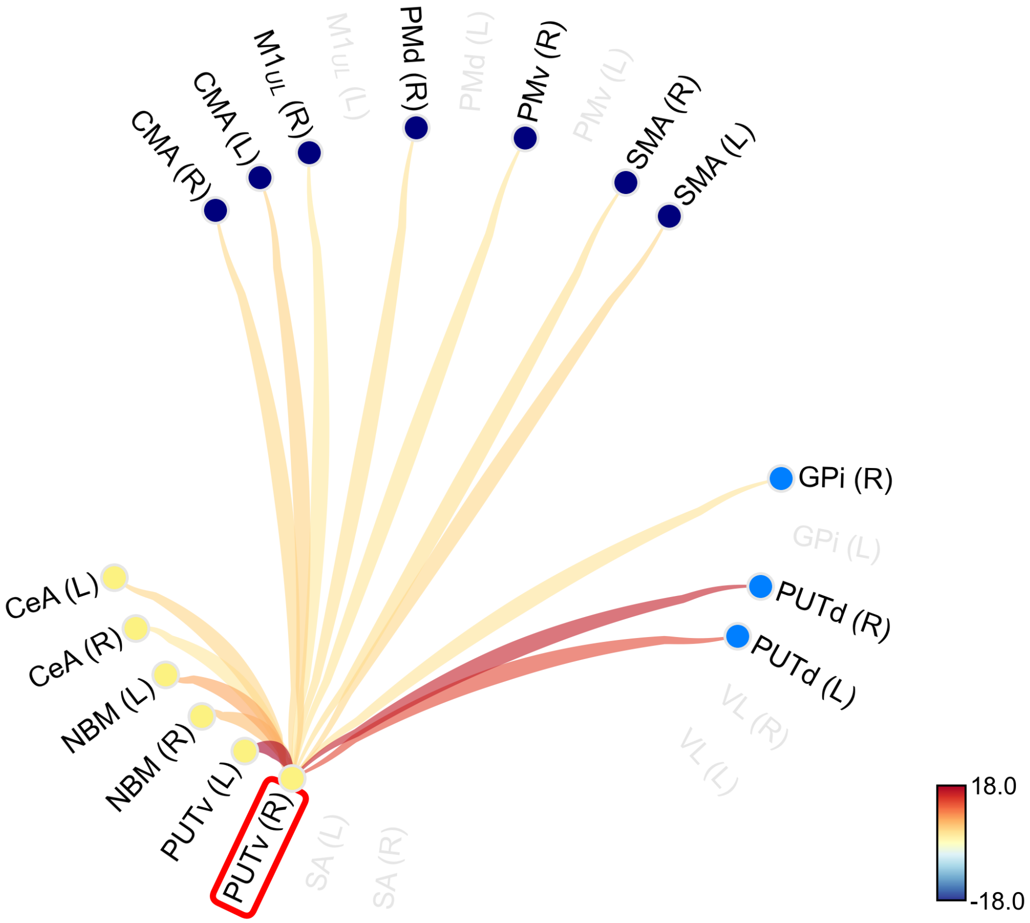

Figure S7 Connectome plot using seed in right ventral putamen; partial correlation (variance from basolateral amygdala removed)

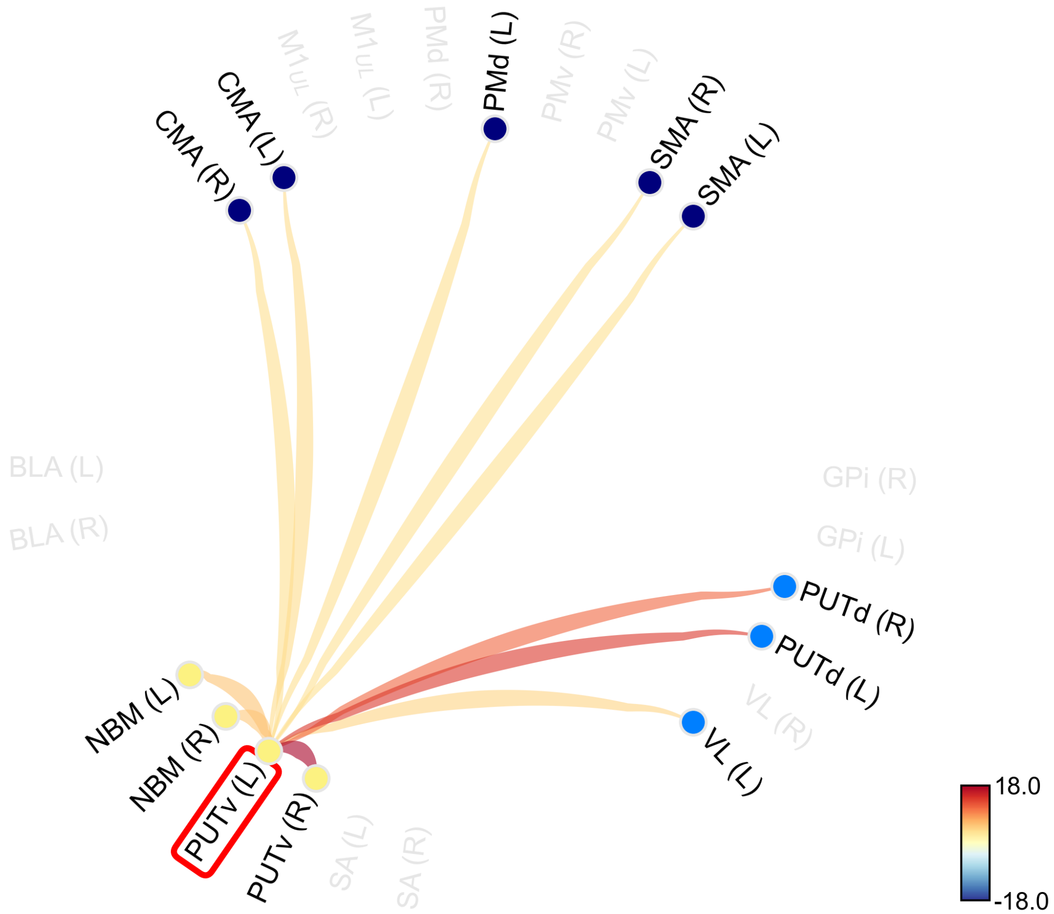

Figure S8 Connectome plot using seed in left ventral putamen; partial correlation (variance from central nucleus of amygdala removed)

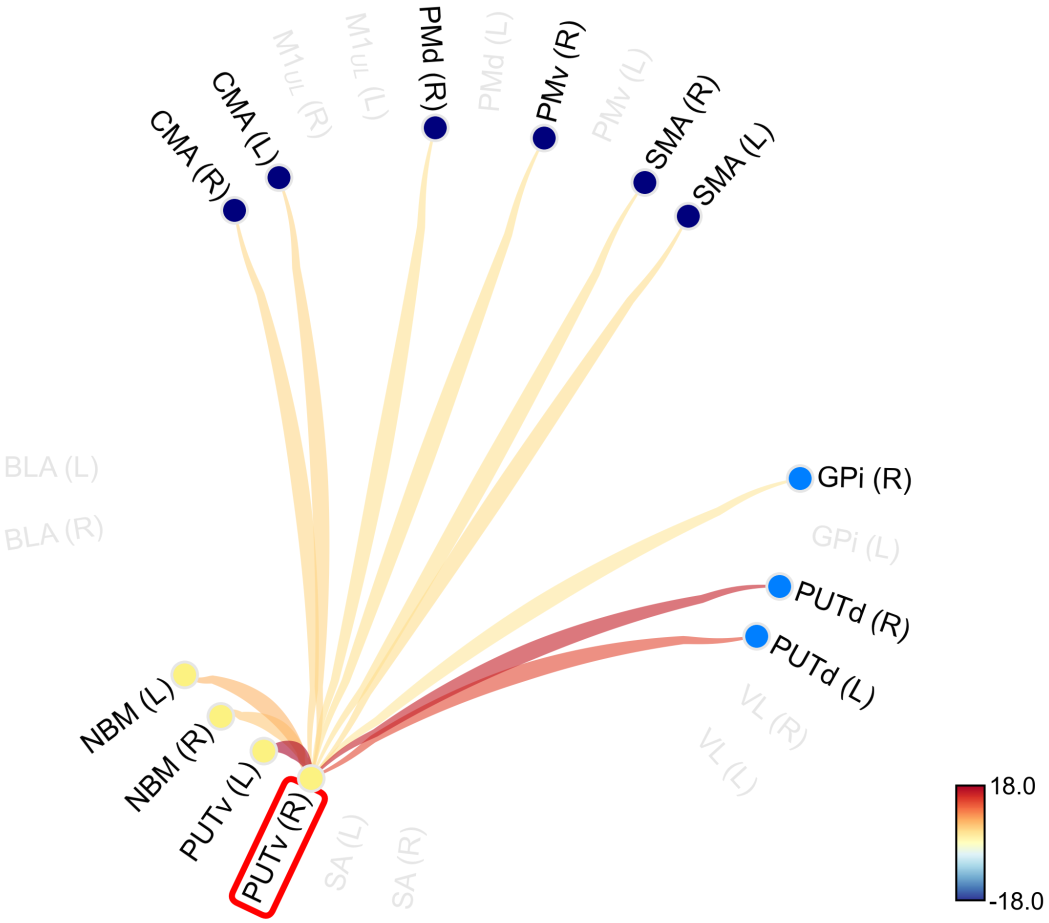

Figure S9 Connectome plot using seed in right ventral putamen; partial correlation (variance from central nucleus of amygdala removed)

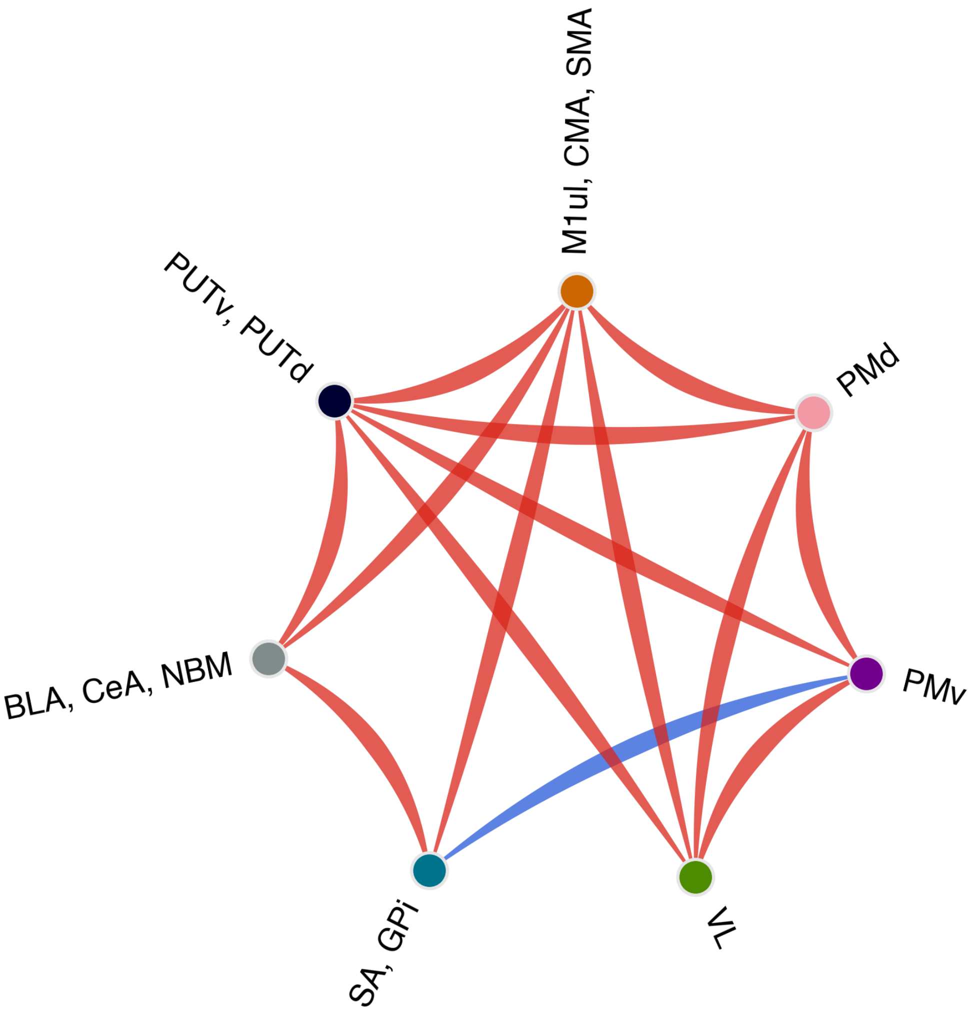

Figure S10 Connectome plot of model-free cluster connectivity analysis of left-hemisphere ROIs. Note that individually-colored circles indicate ROI groups created with model-free hierarchical clustering (i.e., not defined a-priori). A connecting line between two groups (circles) indicates that those groups are significantly connected to one another (red line = positive relationship, blue line = negative). Methods described in SI Methods S3.

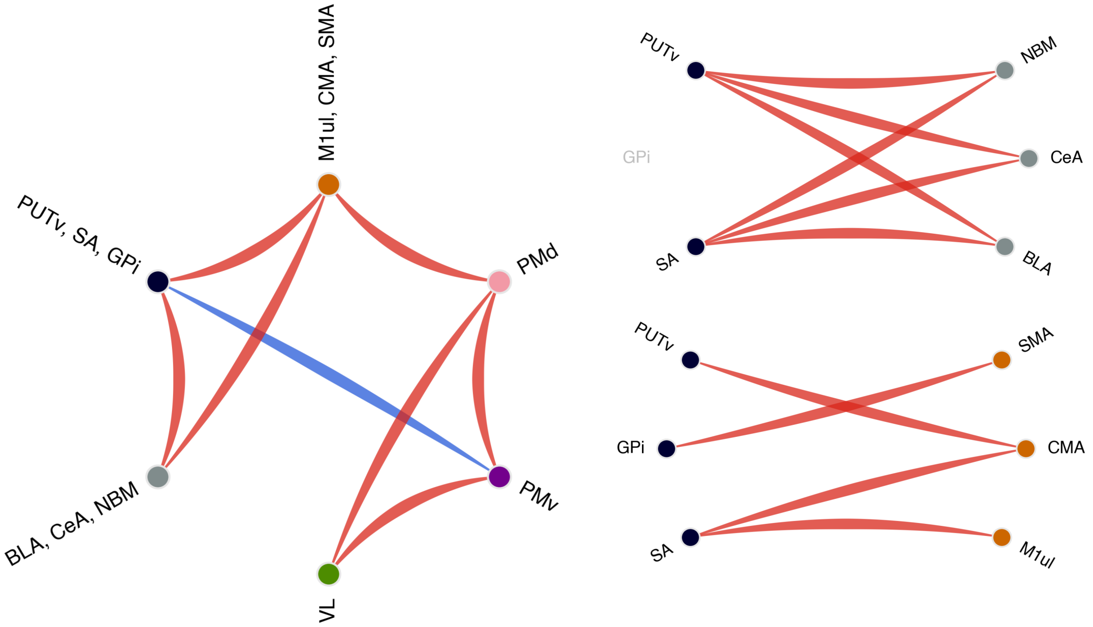

Figure S11 Connectome plot of model-free cluster connectivity analysis of left-hemisphere ROIs (variance from dorsal putamen removed). Left: ROIs are grouped via hierarchical clustering (individually-colored circles). Significant connectivity between groups is indicated by connecting lines (red line = positive relationship, blue line = negative). Top Right: Individual connections that drive significant cluster connectivity between the group containing PUTv (Black) and affective areas (Grey). Bottom Right: Connections that significantly drive connectivity between the group containing PUTv (Black) and cortical motor areas (Orange). Note that connectivity between Black and Orange groups is partially explained by PUTv connectivity with CMA, echoing our results described in the main text. Methods described in SI Methods S3.

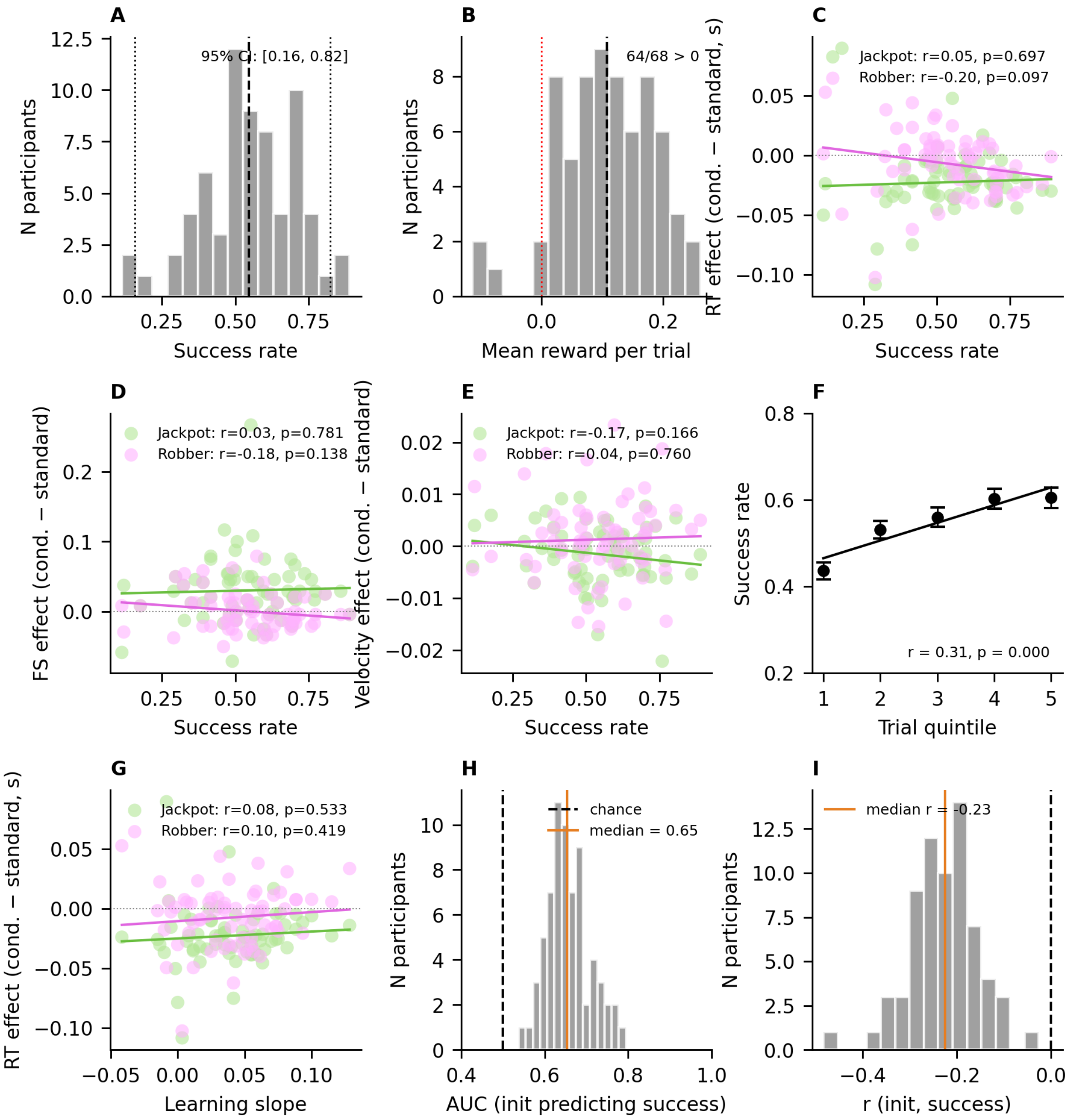

Figure S12 Task success variance across participants and over the task (A, B, F; error bars in F denote SEM across participants), relationship between task success and incentive modulation of RT, false starts, and peak velocity (C-E), relationship between learning slope and RT modulation (G), and coupling between movement initiation speed and trial outcome (H-I). For H-I, point-biserial correlations and logistic regression (AUC) between initiation speed and binary trial outcome (success vs. failure) were computed within each participant; false-start trials were excluded.

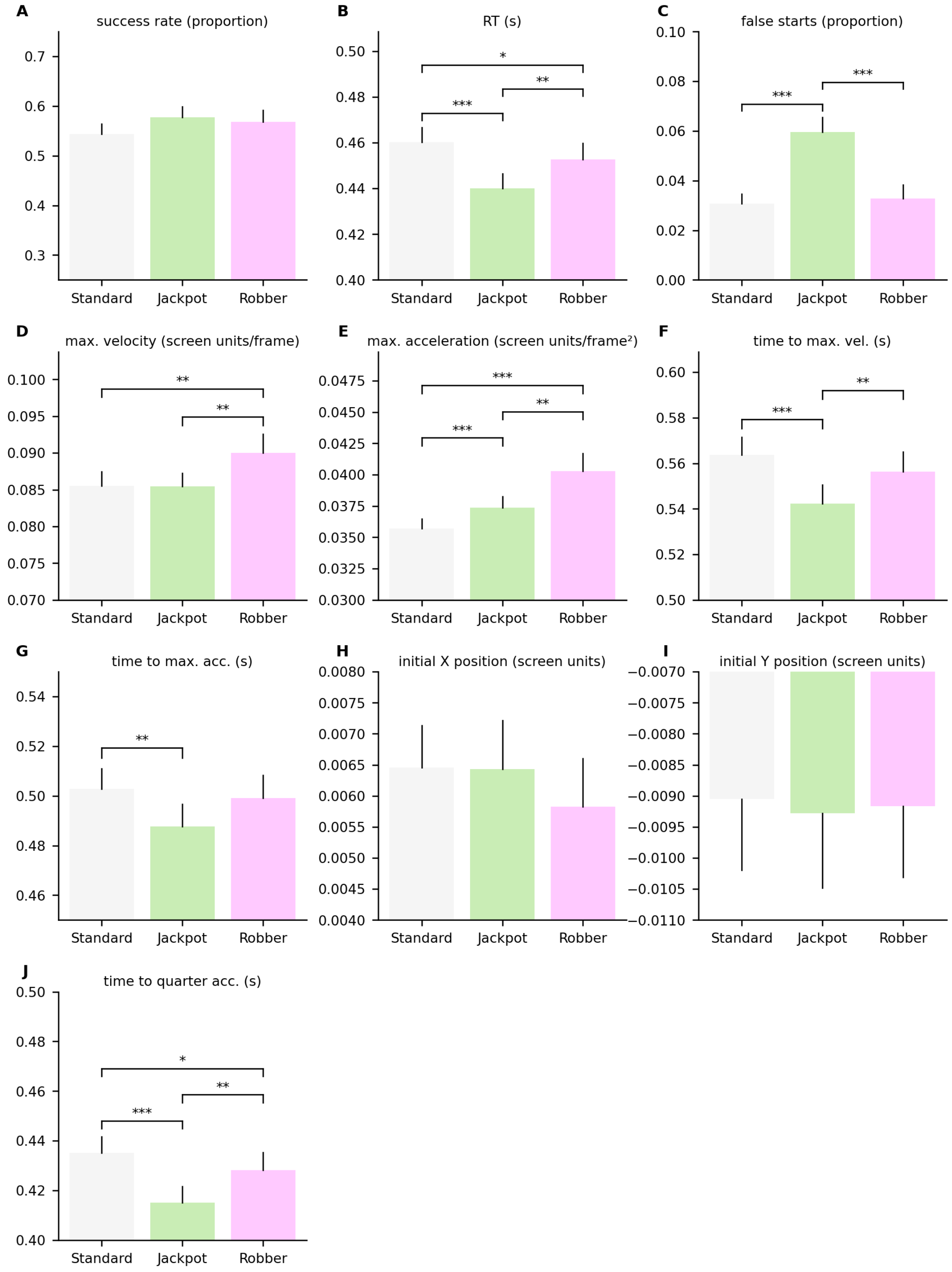

Figure S13 Kinematic variables for each incentive condition. All measures are median per subject, and mean across subject, except proportion success and proportion false start. Figure annotated with pairwise comparison (FDR corrected) if main effect of incentive significant from ANOVA; full results in SI Table S3

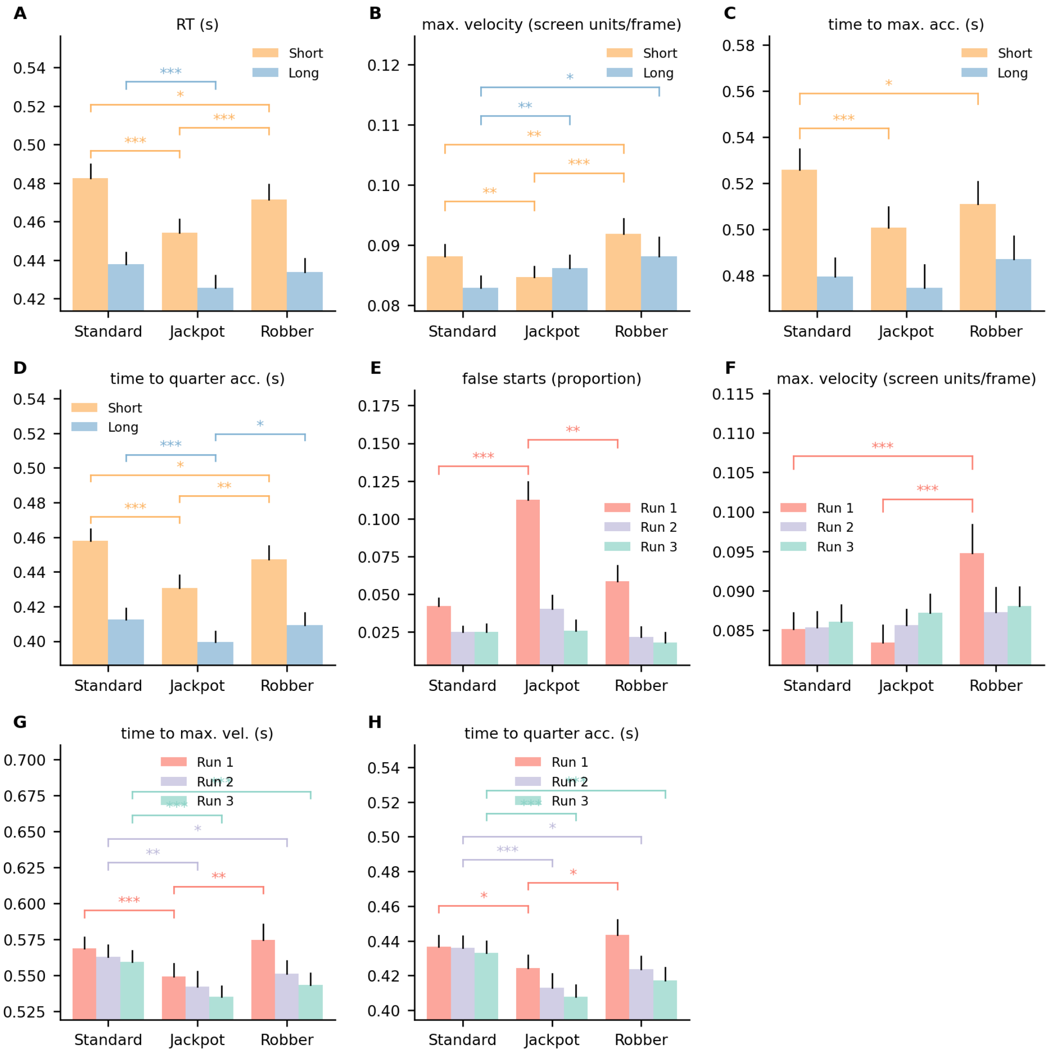

Figure S14 Interaction drilldowns for incentive effects modulated by hold-period duration or run. Panels are included for variables where both the main effect of incentive condition and the relevant two-way interaction (incentive x hold or incentive x run) reached significance after Greenhouse-Geisser correction (see SI Table S3). (A–D) Incentive x hold-period duration: (A) RT, (B) max. velocity, (C) time to max. acceleration, (D) time to quarter acceleration. (E–H) Incentive x run: (E) false starts, (F) max. velocity, (G) time to max. velocity, (H) time to quarter acceleration. Bars show group means; error bars show SEM. Significance markers denote pairwise comparisons between incentive conditions within each moderator level (paired t-tests, Benjamini-Hochberg corrected).

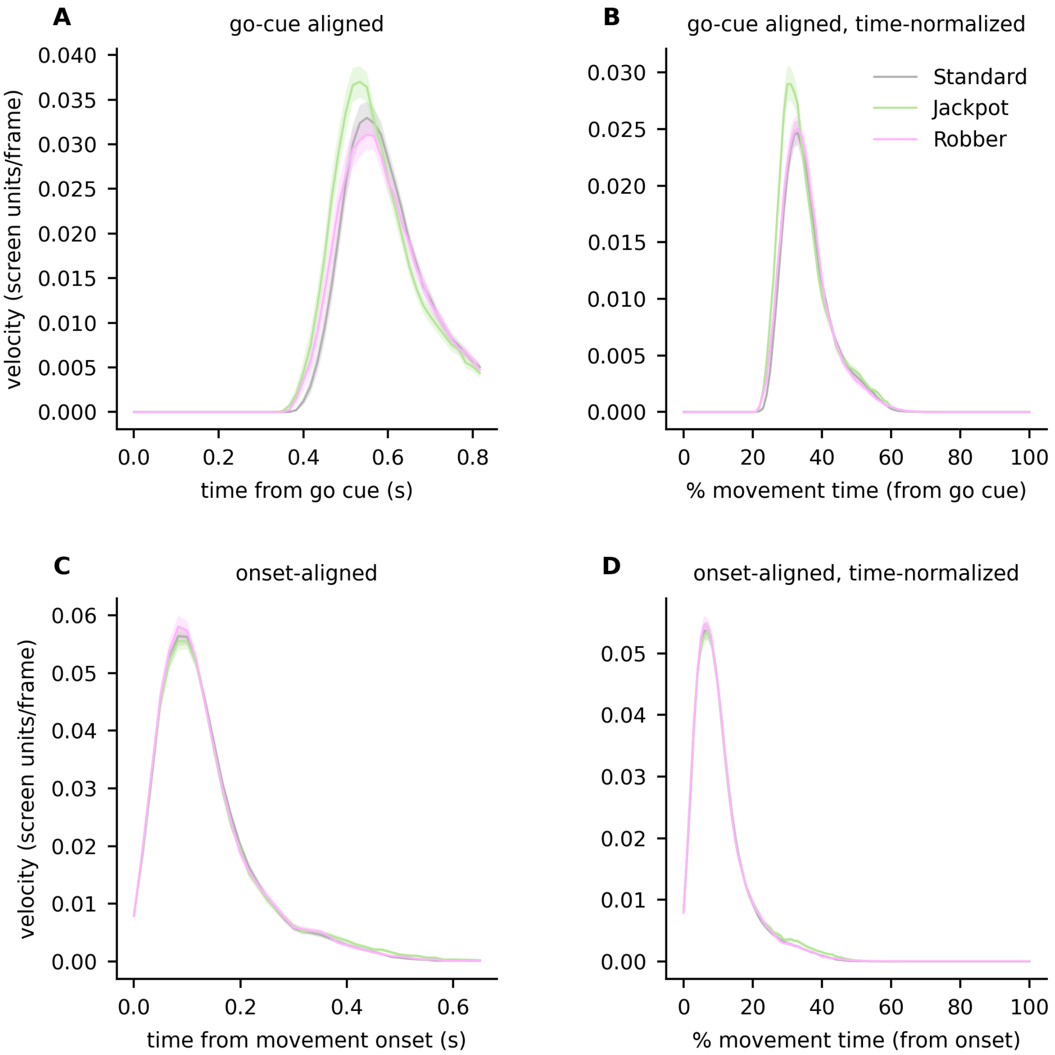

Figure S15 Velocity traces by incentive condition, aligned to the go cue (A, B) or to movement onset (C, D), shown in real time (A, C) or normalized to percentage of movement time (B, D). Radial velocity was computed as the frame-to-frame change in Euclidean distance from the start position (screen units/frame at 60 Hz). Movement onset was defined as the first frame at which tangential displacement exceeded 0.01 screen units. For each panel, trials were first aggregated within participant (nanmedian), then averaged across participants (N = 68); shaded regions denote SEM.

SI Methods

S1. Experiment 1: Participants and Study Protocol

A total of 28 young, healthy participants (15M/13F, mean age = 29.68 years; SD=4.03, all right-handed) completed all study recruitment, pre-screening, and protocol procedures. Study inclusion criteria required that participants be between the ages of 18 − 40 and competent with English enough to understand instructions. Exclusionary criteria were contraindications to MRI (non-removable metal, permanent mouth retainers/braces, incompatible medical devices, vertigo, dizziness, hearing loss/tinnitus, claustrophobia), pregnancy, or clinically significant cardiac, neurological, pulmonary, or psychiatric diseases. Participants were recruited via word of mouth and digital flyers sent to the University of California, Santa Barbara and University of Southern California communities. To assess eligibility, participants completed online screening forms that assessed demographics, health history, and MRI safety eligibility. All participants provided written informed consent for study procedures approved by the Institutional Review Boards at University of California, Santa Barbara and the University of Southern California and were paid 20 U.S. dollars/hour for the full MRI session. Data were acquired at the Center for Image Acquisition at the University of Southern California Mark and Mary Stevens Neuroimaging and Informatics Institute.

On the day of their study session, participants completed the Edinburgh Handedness Inventory [9] and a comprehensive questionnaire assessing demographics, medication use, and health history. Participants then completed a 30-minute MRI protocol at the University of Southern California's Center for Image Acquisition, where they were scanned with a Siemens 7T Terra scanner and a research-only 8-channel transmit 32-channel receive Nova 8Tx/32Rx head coil (Nova Medical Inc.) First, high-resolution T1-weighted magnetization prepared rapid gradient echo (MPRAGE) anatomical scans were acquired (TR = 4300 ms, TE = 2.27 ms, FOV = 240 mm, T1 = 1000 ms, flip angle = 4°, with 0.75 mm^3 voxel size). Following the anatomical scan, modified CMRR multi echo resting-state echo planar imaging (EPI) sequences were acquired (Total time = 10:52 mins, TR = 2100ms, flip angle = 70°, 1.5mm thick axial slices, 2x2 mm in plane, 300 volumes, 200mm FOV, iPat GRAPPA factor = 3, MB factor = 4, phase encoding = posterior to anterior), where participants were instructed to keep their eyes open. The echo times and slice number were slightly modified for the first 8 participants (TE1=15.20ms, TE2=34.23ms, TE3=53.26ms, 84 slices) vs. the remaining 13 (TE1=15.20ms, TE2=33.87ms, TE3=52.54ms, 100 slices). Two spin-echo EPI sequences for field map generation were also acquired: one with anterior to posterior (AP) and another with posterior to anterior (PA) phase encoding to use for distortion correction (TR = 3000ms, TE = 60ms, flip angle = 70°, 1.5mm thick axial slices, 2x2mm in plane, 192mm FOV, iPat GRAPPA factor = 2, MB factor = 5). During scanning, participant respiration and pulse measurements were acquired with an in-scanner Siemens respiration belt and pulse sensor for later denoising.

S2. Experiment 1: Multi-Echo fMRI Preprocessing and Denoising with Tedana

We conducted MRI preprocessing with a custom pipeline featuring Advanced Normalization Tools (ANTs) [10], FSL [11], and TE Dependent ANAlysis version 24.0.1 [12]. Our preprocessing pipeline was inspired by [13]. First, T1-weighted anatomical data were skull-stripped with antsBrainExtraction.sh and resampled to functional image resolution (2mm voxels). We then used FSL Topup with AP and PA spin echo sequences to obtain an undistorted magnitude image and fieldmap for distortion correction. Next, we averaged the EPI data single band reference images at each timepoint across 3 echoes; this reference image was then brain extracted with FSL BET. EPI data were motion-corrected with FSL MCFLIRT (transforms for echo 1 were applied to echoes 2 and 3) and slice time corrected with FSL slicetimer. We used FSL's epi reg with the single-band reference brain to obtain fieldmap distortion correction transforms and co-registration transforms to T1-MPRAGE data. These transforms were then applied to EPI data to place all three echoes into participant-specific anatomical space. Due to fieldmap scan acquisition error, the distortion correction transforms were not applied to the first 12/28 participants.

Next, we denoised resting-state EPI data in anatomical space with Tedana (see Tedana program output below for full details). Briefly, Tedana optimally combines the three echo images, reduces data dimensionality with principal component analysis (PCA), and removes TE-independent (non-BOLD) components from the data with independent component analysis (ICA). After Tedana denoising, we spatially normalized all participant data by registering participant T1-anatomical images to the MNI-152 2mm template and

applying this transformation to the denoised EPI data. Normalized EPI data were then inputted into the CONN Toolbox ([14]; see S3. CONN Functional Connectivity Analysis).

Tedana program output:

Following standard preprocessing, multi-echo EPI data were denoised using TE-dependent analysis (tedana workflow v24.0.1). The ICA decision tree employed was tedana_orig, structurally analogous to the MEICA v2.5 criteria described by [15] and detailed by [16]. A user-defined mask was initially applied, after which an adaptive mask was generated using the dropout method. In this approach, each voxel's value reflects the number of echoes containing valid signal. A two-stage masking procedure was then implemented: a liberal mask (voxels with signal in at least the first echo) was used for optimal combination, T2*/S0 estimation, and denoising, while a conservative mask (restricted to voxels with signal in at least the first three echoes) was applied during component classification.

A monoexponential decay model was fit to each voxel's signal using nonlinear optimization to estimate T2* and S0 maps, with initial values derived from log-linear fits. The adaptive mask determined which echoes contributed to each voxel's parameter estimation. When nonlinear fitting failed, log-linear estimates were retained. Multi-echo data were optimally combined using the T2*-weighted method [17]. Global signal regression was applied to both multi-echo and optimally combined datasets before dimensionality reduction.

Principal component analysis with pre-specified component count was applied to the optimally combined data. The following metrics were computed at this stage: kappa (TE-dependence), rho (TE-independence), countnoise, countsigFT2, countsigFS0, dice_FT2, dice_FS0, signal-noise_t, variance explained, normalized variance explained, and d_table_score. Kappa and rho quantify the degree to which components track T2* decay (BOLD-like) versus remain invariant across TEs (non-BOLD artifacts). A t-statistic (signal-noise_z) and associated p-value (signal-noise_p) were derived by contrasting T2*-model F-statistics between cluster voxels (signal) and non-cluster voxels (noise), measuring component association with signal over noise. The count of significant non-cluster voxels was also tallied per component.

Independent component analysis then decomposed the dimensionally reduced data. Component-level metrics identical to those from PCA were recomputed on ICA outputs. Component classification proceeded via the tedana_orig decision tree to identify BOLD (TE-dependent) versus non-BOLD (TE-independent) sources. Rejected components were removed from the optimally combined data, yielding denoised time series for subsequent connectivity analyses. All computations utilized numpy [18], scipy [19], pandas [20, 21], scikit-learn [22], nilearn, nibabel [23], matplotlib [24], and bokeh [25]. Dice similarity indices were computed as described by [26] and [27].

S3. Experiment 1: CONN Functional Connectivity Analysis

Following tedana denoising, residual confounds were addressed using the CONN functional connectivity toolbox (release 22.a; [28]). Anatomical noise components were extracted via CompCor [29] by computing the five largest principal components orthogonal to the mean BOLD signal within each subject's eroded white matter mask, plus the first principal component from eroded CSF. These were combined with 18 RETROICOR regressors (generated separately with the PhysIO Toolbox [30] from pulse and respiration data) and additional linear trends within each run. Temporal filtering applied a bandpass of 0.008–0.09 Hz to the denoised BOLD time series, following recommendations by [31] to avoid reintroducing noise via spectral misspecification. The effective degrees of freedom post-denoising, accounting for all confound regressors, were estimated to range from 60.6 to 92.3 (mean 88.0) across participants.

Seed-to-voxel connectivity was characterized using bivariate correlation coefficients estimated via weighted general linear model [32]. To compensate for transient magnetization at run onset, individual volumes were weighted by a step function convolved with the SPM canonical hemodynamic response and rectified. Fisher-transformed correlation maps were generated for each seed ROI. ROI-level inference combined connection-level statistics across all voxels projecting from each seed using multivariate parametric tests with random effects across subjects [33]. Significant connections were identified using false discovery rate correction (Benjamini-Hochberg procedure) applied across the upper triangle of the connectivity matrix to avoid double-counting symmetric connections, following the recommended CONN toolbox approach. The FDR threshold was set at $\alpha = 0.05$. Connection strengths are reported as t-statistics from the correlation analysis [34, 35].

Partial correlation analyses isolated PUTv-specific connectivity by regressing the ipsilateral PUTd mean time series from the PUTv seed prior to connectivity estimation. This orthogonalization removed shared variance attributable to canonical motor circuitry. Seed-to-voxel maps were recomputed using the residualized PUTv signal, with identical statistical thresholding applied. Network-level comparisons extracted mean connectivity values across all voxels within anatomically defined target ROIs. Motor network ROIs comprised CMA, SMA, PMd, $M1_{UL}$, and PMv; affective network ROIs comprised BLA, CeA, SA, and NBM. Repeated-measures ANOVA tested for putamen subregion by target network interactions, with Bonferroni correction applied to post-hoc pairwise comparisons. All analyses were conducted in CONN (RRID:SCR_009550) and SPM12 (v7487; Wellcome Centre for Human Neuroimaging).

Model-free analyses (Figs. S10-S11): For each individual connection a separate GLM was estimated, with first-level connectivity measures at this connection as dependent variables (one independent sample per subject and one measurement per task or experimental condition, if applicable), and groups or other subject-level identifiers as independent variables. Connection-level hypotheses were evaluated using multivariate parametric statistics with random-effects across subjects and sample covariance estimation across multiple measurements. Inferences were performed at the level of individual clusters (groups of similar connections). Cluster-level inferences were based on parametric statistics within- and between- each pair of networks (Functional Network Connectivity; [36]), with networks identified using a complete-linkage hierarchical clustering procedure [27] based on ROI-to-ROI anatomical proximity and functional similarity metrics [35]. Results were thresholded using a combination of a $p < 0.05$ connection-level threshold and a familywise corrected p-FDR < 0.05 [34].

S4. Experiment 2: Participants and Study Protocol

A total of 68 young, healthy participants (50F/18M, mean age = 20.75 years; SD = 1.86) completed all study recruitment, pre-screening, and protocol procedures. Study inclusion criteria required that participants be between the ages of 18–40 and competent with English enough to understand instructions. Exclusionary criteria were contraindications to MRI (non-removable metal, permanent mouth retainers/braces, incompatible medical devices, vertigo, dizziness, hearing loss/tinnitus, claustrophobia), pregnancy, or clinically significant cardiac, neurological, pulmonary, or psychiatric diseases. Participants were recruited via word of mouth and a study recruitment portal at the University of California, Santa Barbara. To assess eligibility, participants completed online screening forms that assessed demographics, health history, and MRI safety eligibility. All participants provided written informed consent for study procedures approved by the Institutional Review Board at University of California, Santa Barbara.

Each subject's data were collected in a single session, lasting approximately two hours. fMRI data were recorded using a Siemens 3T Prisma scanner with a 64-channel phased-array head/neck coil. Task responses were recorded using an MR-compatible joystick. This was fixed to the center of a wooden table, which was placed across participants' torsos once they were supine in the scanner bore. The position of the table was adjusted along the bore to ensure a comfortable distance for the participant's arm. Arms were supported by pillows and padding.

Each session began with a T1-weighted magnetization prepared rapid gradient echo (MPRAGE) anatomical scan (TR = 2500ms, TE = 2.22ms, FOV = 241mm, T1 = 851ms, flip angle = 7°, $0.94mm^3$ voxel size). We next acquired a double-echo gradient echo field map sequence for distortion correction (TR = 758 ms, TE = 4.92ms, flip angle = 60°, $2.5mm^2$ thick axial slices). We then acquired 4-dimensional echo-planar imaging during each of the three runs of the task (TR = 1900 ms, TE = 30 ms, flip angle = 65°, $2.5mm^3$ voxel size, multiband acceleration factor of 2).

S5. Experiment 2: Incentivized Vigor Task

Participants performed a speeded precision joystick reaching task in which they moved a cursor to a cued target under one of three incentive conditions: jackpot ($1.60 reward), robber ($1.60 loss avoidance), or standard ($0.20 reward). Joystick displacement was position-mapped to screen cursor position, with full deflection of the joystick corresponding to the edge of the display. Both targets were positioned well within this range (see below). This mapping meant that participants could overshoot or undershoot the target during the reach, requiring online deceleration and correction to achieve and maintain the hold criterion.

The task thus demands both rapid initiation and precise online control for successful completion. The task comprised 300 trials (10% jackpot, 10% robber, 80% standard) across three runs.

The deadline for reach completion from onset of the go cue (1.87s) was calibrated from a pilot sample to achieve a 50% success rate across participants. This pilot sample of eight participants performed the task inside the scanner environment. From their data we used Bayesian estimation of the group-level mean and standard deviation of log-transformed completion times to infer the time associated with a 50% probability of completion.

Visual stimuli were programmed in PsychoPy [37] and rear-projected onto a screen placed approximately 110cm behind participants using an LCD projector (1920×1080, 60 Hz). Participants viewed rectified images by way of a double mirror in the head coil. Each trial began once participants held a screen cursor (radius=0.5°; yoked to joystick position) at a starting position (radius=1.2°; ∼9.5° below screen center). An instruction cue then appeared at one of two spatial locations. Each location had a radius of 1.2° and were both ∼9.5° above the screen center. The left target was ∼19° to the left of the screen's midline, and the right target ∼19° to the right. Each instruction cue was made of the same small blue squares, re-arranged to make a neutral (standard trial), happy (jackpot) or sad (robber) face. Each stimulus was ∼6.6° radius and appeared centered on one of the two spatial targets for 200 ms. Once participants held the cursor at the starting position, a "wait" period (1 or 2 s) elapsed before the instruction cue appeared. Following the instruction cue, a "hold" period (1, 2, 3, or 4 s) elapsed before the go cue. These durations were assigned on each trial according to a modified m-sequence (wait: 1, n=162; 2, n=138; hold: 1, n=96; 2, n=75; 3, n=66; 4, n=63). Total completion time on each trial was the time from the onset of the go cue until they reached the cued target, holding it in place for an additional 0.8 s. Completions within the deadline (1.87 s) were successful, with verbal feedback indicating the trial's reward alongside the word "success". Completions slower than this deadline were unsuccessful, with verbal feedback indicating the trial's loss alongside the word "too slow". All text was ∼2° in height and appeared at the screen center. Participants performed a set of 40 training trials prior to the experimental runs, where difficulty gradually increased from a deadline of 5s to the task target.

Cursor position was continuously recorded on each frame. Movement initiation time (RT) was defined as the time taken to initiate movement (cursor moving outside starting position, i.e., centroid exceeding radius of the starting position) relative to the onset of the go-cue. False-starts were any movement of the cursor outside of the starting position after the onset of the instruction cue, prior to the onset of the go cue. Behavioral analyses of success rate, reaction time, false starts, maximum velocity, maximum acceleration, time to maximum velocity, time to maximum acceleration, initial cursor position, and time to quarter acceleration were conducted with repeated-measures ANOVA using factors of incentive condition (standard, jackpot, robber), hold period (1 or 2 s (short) vs. 3 or 4 s (long)) and run (1,2,3), and all interactions. Two-way interactions are reported to assess whether incentive effects were modulated by hold-period duration or run; three-way interactions were estimated but not reported absent a priori predictions. Greenhouse-Geisser correction was applied to all effects with numerator degrees of freedom greater than one. Post-hoc pairwise comparisons used paired t-tests corrected with the Benjamini-Hochberg false discovery rate (FDR) procedure.

S6. Experiment 2: Task-Based fMRI Preprocessing, Modeling, and Bayesian Inference

We conducted MRI preprocessing with a custom pipeline featuring Advanced Normalization Tools [10] and FSL [11] on 62 of the 68 participants showing jackpot effects in RT. T1-weighted anatomical data were skull-stripped with antsBrainExtraction.sh and resampled to functional image resolution (2mm voxels). We then applied motion, slicetime, and distortion correction with field maps to EPI data with FSL FEAT. Next, we used ANTs (antsRegistrationSyN) to coregister and apply a non linear transform of EPI data to respective T1 anatomical data, then to MNI152 2mm space. EPI data were smoothed with a 5 mm FWHM Gaussian kernel.

First-level GLMs were fitted to preprocessed EPI data using FSL FEAT. The model included seven regressors of interest plus two nuisance regressors. Instruction-phase regressors were modeled as stick functions (unit height, 0.1 s width) event-locked to cue onset, separately for standard, jackpot, and robber trials. Go-phase regressors were duration-scaled and event-locked to go cue onset, also separated by incentive condition.

For a trial with initiation time (RT) of t seconds, the corresponding go-phase regressor had duration t and unit height. Under this parametrization, slower initiation (longer RT) produces longer regressor duration, such that positive regression coefficients indicate greater BOLD signal with slower movement initiation. For interpretability, plotted values are sign-reversed so that positive values reflect greater BOLD activity associated with faster initiation.

Nuisance regressors captured task-related activity not specific to RT effects. The pre-go hold regressor had onset at instruction cue offset and duration equal to the hold period (1–4 s, varied by trial). The reach execution regressor had onset at movement initiation (RT from go cue) and duration from initiation to trial end. The reach execution regressor thus captures variance associated with the reach itself, target hold, and feedback display, and is intended to account for outcome-related activity, reducing its contribution to the go-phase engagement estimate. Both nuisance regressors were unit height and duration-scaled. Additional confound regressors for motion parameters and temporal derivatives were included following standard FSL procedures.

Run-level parameter estimates (cope files) for each regressor were extracted and averaged within subjects to create subject-level summaries. For each ROI, mean activation across all voxels was computed per subject and regressor, yielding a single value per subject, per ROI, per contrast. These ROI-averaged values were then submitted to group-level Bayesian inference.

Hierarchical Bayesian models were implemented to estimate group-level activation parameters while accounting for between-subject variability and accommodating outliers. For each ROI and contrast combination, the distribution of subject-level activations was modeled as a Student's t distribution with location parameter μ, scale parameter σ, and degrees-of-freedom parameter ν. A single mixture model was fitted across all ROI-contrast pairs simultaneously. Uninformed priors were assigned: $\mu_{\text{ROI,contrast}} \sim \mathcal{N}(0, 1)$, $\sigma_{\text{ROI,contrast}} \sim \text{HalfNormal}(1)$, and a single $\nu \sim \text{HalfNormal}(1)$ was shared across all distributions to pool information about outlier prevalence.

Posterior distributions were sampled using Markov Chain Monte Carlo (MCMC). Four chains were run for 2000 tuning iterations followed by 2000 sampling iterations. Convergence was assessed via $\hat{R} < 1.01$ for all parameters. Circuit-level estimates were derived by averaging node-level posterior draws at each MCMC iteration: for circuit c in incentive condition x, $\mu_{x,c} = \frac{1}{N}\sum_{i=1}^{N} \mu_{x,i}$ where i indexes the N nodes in circuit c. This approach propagates uncertainty from node-level to circuit-level estimates. Activations were deemed credibly nonzero when the 89% highest-density interval (HDI) of the posterior distribution excluded zero. Contrasts between circuits or incentive conditions were evaluated by computing posterior differences ($\Delta = \mu_1 - \mu_2$) at each MCMC draw and assessing whether the resulting HDI excluded zero.

All Bayesian analyses were conducted in Python 3.9.21 using PyMC3 v3.11.2 for sampling [38], ArviZ v0.12.1 for diagnostics [39], and NumPy v1.22.3 for numerical operations [18]. Full model specifications and convergence diagnostics are available in the analysis code repository.

SI References

[1] Tyszka, J. M., & Pauli, W. M. (2016). In vivo delineation of subdivisions of the human amygdaloid complex in a high-resolution group template. *Human Brain Mapping*, 37(11):3979–3998.

[2] Di Martino, A., et al. (2008). Functional Connectivity of Human Striatum: A Resting State fMRI Study. *Cerebral Cortex* 18(12):2735–2747

[3] Zaborszky, L., et al. (2008). Stereotaxic probabilistic maps of the magnocellular cell groups in human basal forebrain. *NeuroImage*, 42(3):1127–1141.

[4] Amunts, K., et al. (2020). Julich-Brain: A 3D probabilistic atlas of the human brain's cytoarchitecture. *Science*, 369(6506):988-992.

[5] Pauli, W. M., et al. (2018). A high-resolution probabilistic in vivo atlas of human subcortical brain nuclei. *Scientific data*, 5(1):180063.

[6] Fan, L., et al. (2016). The Human Brainnetome Atlas: A New Brain Atlas Based on Connectional Architecture. *Cerebral Cortex*, 26(8):3508–3526.

[7] Paus, T. (2001). Primate anterior cingulate cortex: Where motor control, drive and cognition interface. *Nature Reviews Neuroscience*, 2(6):417–424. https://doi.org/10.1038/35077500

[8] Desikan, R. S., et al. (2006). An automated labeling system for subdividing the human cerebral cortex on MRI scans into gyral based regions of interest. *NeuroImage*, 31(3):968–980.

[9] Oldfield RC (2013) Edinburgh Handedness Inventory. https://doi.org/10.1037/t23111-000.

[10] Avants BB, et al. (2011) A reproducible evaluation of ANTs similarity metric performance in brain image registration. *NeuroImage* 54:2033–2044.

[11] Jenkinson M, et al. (2012) FSL. *NeuroImage* 62:782–790.

[12] DuPre E, et al. (2021) TE-dependent analysis of multi-echo fMRI with tedana. *J Open Source Softw* 6(66):3669.

[13] Lynch CJ, et al. (2020) Rapid precision functional mapping of individuals using multi-echo fMRI. *Cell Reports* 33:108540.

[14] Whitfield-Gabrieli S, Nieto-Castanon A (2012) Conn: a functional connectivity toolbox for correlated and anti-correlated brain networks. *Brain Connectivity* 2:125–141.

[15] Kundu P, et al. (2013) Integrated strategy for improving functional connectivity mapping using multiecho fMRI. *Proc Natl Acad Sci USA* 110(40):16187–16192.

[16] Olafsson V, et al. (2015) Enhanced identification of BOLD-like components with multi-echo simultaneous multi-slice fMRI and multi-echo ICA. *NeuroImage* 112:43–51.

[17] Posse S, et al. (1999) Enhancement of BOLD-contrast sensitivity by single-shot multi-echo functional MR imaging. *Magn Reson Med* 42(1):87–97.

[18] Van Der Walt S, Colbert SC, Varoquaux G (2011) The NumPy array: a structure for efficient numerical computation. *Comput Sci Eng* 13(2):22–30.

[19] Virtanen P, et al. (2020) SciPy 1.0: fundamental algorithms for scientific computing in Python. *Nat Methods* 17(3):261–272.

[20] McKinney W (2010) Data structures for statistical computing in Python. *Proc 9th Python Sci Conf*, pp 51–56.

[21] pandas development team (2020) *pandas-dev/pandas*: Pandas 1.0.1. doi:10.5281/zenodo.3644238

[22] Pedregosa F, et al. (2011) Scikit-learn: machine learning in Python. *J Mach Learn Res* 12:2825–2830.

[23] Brett M, et al. (2019) *nipy/nibabel* 2.4.1. doi:10.5281/zenodo.3233118

[24] Hunter JD (2007) Matplotlib: a 2D graphics environment. *Comput Sci Eng* 9(3):90–95.

[25] Bokeh Development Team (2018) *Bokeh: Python library for interactive visualization*. Available at: https://bokeh.pydata.org

[26] Dice LR (1945) Measures of the amount of ecologic association between species. *Ecology* 26(3):297–302.

[27] Sorensen TA (1948) A method of establishing groups of equal amplitude in plant sociology based on similarity of species content. *Biol Skar* 5:1–34.

[28] Nieto-Castanon A (2022) *CONN functional connectivity toolbox* (RRID:SCR_009550), release 22.a. doi:10.56441/hilbertpress.2246.5840

[29] Behzadi Y, et al. (2007) A component based noise correction method (CompCor) for BOLD and perfusion based fMRI. *NeuroImage* 37(1):90–101.

[30] Kasper L, et al. (2017) The PhysIO toolbox for modeling physiological noise in fMRI data. *Journal of Neuroscience Methods* 276:56–72.

[31] Hallquist MN, et al. (2013) The nuisance of nuisance regression: spectral misspecification in a common approach to resting-state fMRI preprocessing reintroduces noise and obscures functional connectivity. *NeuroImage* 82:208–225.

[32] Nieto-Castanon A (2020a) Functional connectivity measures. In: *Handbook of fMRI Methods in CONN*, pp 26–62. Hilbert Press.

[33] Nieto-Castanon A (2020b) General linear model. In: *Handbook of fMRI Methods in CONN*, pp 63–82. Hilbert Press.

[34] Benjamini Y, Hochberg Y (1995) Controlling the false discovery rate: a practical and powerful approach to multiple testing. *J R Stat Soc B* 57(1):289–300.

[35] Nieto-Castanon A (2020c) Cluster-level inferences. In: *Handbook of fMRI Methods in CONN*, pp 83–104. Hilbert Press.

[36] Jafri, M. J., et al. (2008). A method for functional network connectivity among spatially independent resting-state components in schizophrenia. *Neuroimage*, 39(4):1666-1681.

[37] Peirce JW, et al. (2019) PsychoPy2: experiments in behavior made easy. *Behav Res Methods* 51(1):195–203.

[38] Salvatier J, et al. (2016) Probabilistic programming in Python using PyMC3. *PeerJ Comput Sci* 2:e55.

[39] Martin OA, et al. (2026) ArviZ: a modular and flexible library for exploratory analysis of Bayesian models. *J Open Source Softw* 11(119):9889.
